# Anemia and Hypoxia Impact on Chronic Kidney Disease Onset and Progression: Review and Updates

**DOI:** 10.7759/cureus.46737

**Published:** 2023-10-09

**Authors:** Elmukhtar Habas, Aisha Al Adab, Mehdi Arryes, Gamal Alfitori, Khalifa Farfar, Ala M Habas, Raza A Akbar, Amnna Rayani, Eshrak Habas, Abdulnaser Elzouki

**Affiliations:** 1 Internal Medicine, Hamad General Hospital, Doha, QAT; 2 Internal Medicine, Tripoli University, Tripoli, LBY; 3 Hemat-oncology Department, Pediatric Tripoli Hospital, Tripoli University, Tripoli, LBY

**Keywords:** hemoglobin level in ckd, chronic kidney disease (ckd), acute kidney injury (aki) and hypoxia, hypoxia, ckd progression, hypoxia physiology, renal hypoxia, anemia in ckd

## Abstract

Chronic kidney disease (CKD) is caused by hypoxia in the renal tissue, leading to inflammation and increased migration of pathogenic cells. Studies showed that leukocytes directly sense hypoxia and respond by initiating gene transcription, encoding the 2-integrin adhesion molecules. Moreover, other mechanisms participate in hypoxia, including anemia. CKD-associated anemia is common, which induces and worsens hypoxia, contributing to CKD progression. Anemia correction can slow CKD progression, but it should be cautiously approached.

In this comprehensive review, the underlying pathophysiology mechanisms and the impact of renal tissue hypoxia and anemia in CKD onset and progression will be reviewed and discussed in detail. Searching for the latest updates in PubMed Central, Medline, PubMed database, Google Scholar, and Google search engines were conducted for original studies, including cross-sectional studies, cohort studies, clinical trials, and review articles using different keywords, phrases, and texts such as “CKD progression, anemia in CKD, CKD, anemia effect on CKD progression, anemia effect on CKD progression, and hypoxia and CKD progression”.

Kidney tissue hypoxia and anemia have an impact on CKD onset and progression. Hypoxia causes nephron cell death, enhancing fibrosis by increasing interstitium protein deposition, inflammatory cell activation, and apoptosis. Severe anemia correction improves life quality and may delay CKD progression. Detection and avoidance of the risk factors of hypoxia prevent recurrent acute kidney injury (AKI) and reduce the CKD rate. A better understanding of kidney hypoxia would prevent AKI and CKD and lead to new therapeutic strategies.

## Introduction and background

Chronic kidney disease (CKD) is a prevalent condition [1]. It is strongly believed that kidney tissue hypoxia is the principal cause of kidney dysfunction. Anemia is commonly associated with CKD [2]. Hemoglobin (Hb) levels of < 13.0 g/dl in men and < 12.0 g/dl in premenopausal women typically indicate anemia, as documented by the World Health Organization (WHO) [3]. CKD-associated anemia is usually normochromic, normocytic, or hypoproliferative, although microcytic hypochromic anemia occurs. It frequently correlates with poor CKD outcomes and increasing mortality [4]. CKD anemia is a multifactorial condition caused by erythropoietin (EPO) deficiency, uremia-induced erythropoiesis inhibition, erythrocyte’s decreased longevity, and iron homeostasis dysregulation [5].

Anemia is a marker of kidney dysfunction; however, the precise relationship between glomerular filtration rate (GFR) and the anemia frequency still needs to be clearly determined [6]. Risk factors of CKD such as hypertension (HTN), diabetes mellitus (DM), proteinuria, and metabolic syndrome (MetS) increased as GFR declined. There is a relationship between anemia prevalence and the estimated GFR (eGFR) [6]. Anemia is uncommon, as the GFR is > 80 ml/min/1.73 m². However, anemia severity increases when the eGFR declines < 60 ml/min/1.73 m² [7]. CKD development, including end-stage renal disease (ESRD), is negatively influenced by anemia [8]. For instance, a four-year study of > 1500 patients with diabetic nephropathy indicated that the adjusted risk of ESRD developing was approximately two times higher in individuals with lower baseline Hb values than in those with the highest baseline Hb values (>13.8 g/dl) [9]. In a study of 415 CKD patients, anemia and left ventricular hypertrophy (LVH) were linked to a faster rate of renal function decline in either one condition alone or with both conditions [8]. Further research has confirmed the link between anemia and fast CKD progression in various groups [10,11]. Although the exact cause of the fast drop in kidney function with more severe anemia is unknown, it might be due to low-grade renal ischemia or the underlying inflammatory effect due to hypoxia, accelerating anemia development and CKD progression. Many methods by which EPO could have effects have been assumed based on animal models of ischemia and nephrotoxic kidney damage. These include decreased caspase activity, enhanced tubular cell regeneration, decreased interstitial fibrosis, and reduced apoptosis [12].

**Figure 1:**
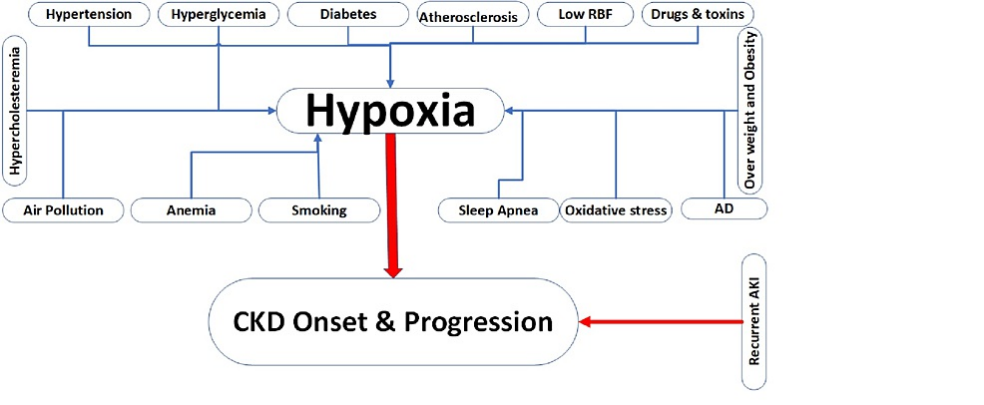
The risk factors of hypoxia cause acute kidney injury and chronic kidney disease. AKI: acute kidney injury; CKD: chronic kidney disease; RBF: renal blood flow; AD: autoimmune diseases The figure is authors’ own creation.

Studies have shown that CKD is caused by renal cortex and medulla hypoxia, which triggers inflammatory reactions and increased leukocyte migration to the hypoxia site. Kidney hypoxia is believed to be caused by cytokines released by the ischemic endothelium. However, recent findings have revealed that leukocyte activation is a response unrelated to endothelial cell hypoxia. Instead, leukocytes have a direct sense of hypoxia and respond by inducing gene transcription that encodes the 2-integrin adhesion molecules. Hypoxia can be due to organ-related causes or hypoxemia. Hypoxia has distinct types (hypoxic, anemic (hypoxemic), stagnant (circulatory), and histotoxic hypoxia). CKD often leads to anemia; however, research suggests that anemia could also contribute to the decline of eGFR directly or by aggravating hypoxia. Furthermore, anemia is a self-determining factor for CKD progression, and correcting anemia could slow CKD progression. However, there are conflicting data about the benefit of complete anemia correction in CKD, and if indicated, it should be started, although it must be carefully considered. Furthermore, erythrocytes contain antioxidants that may help prevent glomerulosclerosis and tubulointerstitial damage caused by oxidative stress, reducing ischemia risk. All the possible related causes of hypoxia that cause kidney damage are illustrated in Figure 1.

Due to the implications of anemia, hypoxia, and the available evidence about anemia and hypoxia as risk factors that have a role in CKD progression, we decided to write this comprehensive nonsystematic review and updates about the relationship between anemia and hypoxia impact on CKD onset, progression, prevention, and controlling these factors how can they reduce CKD onset and progression. As stated in the abstract section, different phrases and keywords were used in searching for articles in Google and PubMed.

## Review

Anemia effects in CKD

Anemia is acknowledged as an independent physiological risk factor for CKD development and progression [13,14]. Anemia is characterized by low Hb content in the circulation, diminishing oxygen-carrying capacity that produces anemic hypoxia, which could impair renal oxygenation. CKD is a major contributor to ESRD, cardiovascular disease (CVD), cancer, and infection. Anemia is prevalent in ESRD and develops early in CKD, increasing the risk of congestive heart failure (CHF), CKD, and LVH. Anemia is counted as a non-conventional factor for CKD progression [6]. Treating anemia with erythropoiesis-stimulating drugs (ESA) improves quality of life and stops the development of CKD. However, different studies have shown that anemia may improve renal and CVD outcomes, and prospective trials have not been able to confirm the positive effects on the survival rate for people with ESRD and CKD stages 3 and 4. Similarly, controlling other variables like hyperlipidemia did not substantially affect the survival of dialysis patients. With higher anemia prevalence in ESRD and its damaging effects on CKD, suitable intervention to improve the anemia is necessary for practicing medical professionals caring for high-risk populations. It is vital to amend renal anemia with low Hb (<10 g/dl), but the Hb level must not be > 13 g/dl in males, and 12 g/dl in women.

In the Epo-TAg^h^ transgenic mouse-induced severe anemia due to a disrupted EPO encoding gene had developed renal dysfunction due to the exhibited renal hypoxia [14,15]. Interestingly, in addition to correcting anemia, EPO administration protects against ischemia-induced kidney injury [16]. However, this safeguarding effect appears to be the renoprotective EPO effect due to mechanisms unrelated to its capability to increase erythropoiesis [17,18]. EPO has a heterodimer EPO receptor and CD131 on the renal cells’ surface, prorogating subsequent intracellular signaling and cascades [17-19]. Apoptosis is inhibited because of several factors’ activation, including, decreased pro-apoptotic nuclear factor B and Bcl-2-like protein 4 expression by these signals due to EPO receptor activation [17,18,20].

EPO contributes to renoprotection by mitigating oxidative stress by upregulating glutathione peroxidase, superoxide dismutase, and endothelial nitric oxide (NO) synthase [17,21]. As a response to ischemia, EPO inhibits renal tissue’s ability to produce high pro-inflammatory cytokines, intercellular adhesion molecules, and chemokines levels. Consequently, renal tissues become more resistant to neutrophil and macrophage attacks [20]. Conflicting data on anemia correction influence the pace at which renal failure advances. Darbepoetin alfa was given to 4038 subjects with type 2 DM and CKD with the eGFR range of 20-60 ml/min/1.73 m^2^) in the Trial to Reduce Cardiovascular Events with Aranesp Therapy (TREAT) to accomplish the target Hb (13 g/dl) or to placebo, with darbepoetin being given when the Hb was < 9 g/dl [22]. The mean attained Hb level in the darbepoetin and placebo groups was 12.5 g/dl and 10.6 g/dl, respectively. After a rigorous follow-up period of 29 months, it was conclusively determined that there is no discernible disparity in ESRD among the groups (16.8 vs. 16.3% in placebo, hazard ratio (HR) 1.02, 95% CI 0.87-1.18). However, a secondary analysis of Hb Correction and Outcomes in Renal Insufficiency (CHOIR) trial reported that the higher Hb target group had a higher rate of initiating renal replacement therapy (RRT) and a higher composite endpoint risk for CKD progression (creatinine doubling, RRT start, or death), and increases the rate of CKD progression [23]. Larger studies of CKD-associated anemia therapy revealed a tendency for CKD progression risk with high Hb goals, according to one meta-analysis; nevertheless, in the total pooled analysis, no difference was seen between higher and lower targets [24]. A meta-analysis report demonstrated that ESA therapy was not significantly different from no treatment or placebo for CKD progression indicators [25]. Two meta-analyses were conducted to compare the efficacy of higher versus lower Hb values and ESA treatment with control in protecting against CKD progression. Two studies demonstrated no significant protective benefit in either case [26,27].

Positive dipstick proteinuria and elevated blood pressure (BP) were significant markers for CKD progression to ESRD predictions, according to a study on predictors of ESRD based on community registries from the general screening program and the dialysis population [27,28]. The baseline hematocrit (Hct) influence on the development of ESRD was also taken into account, demonstrating that low Hct speeds up the evolution of CKD [5,29]. Furthermore, the baseline Hct effect on ESRD development was also considered, proving that low Hct increases CKD progression to ESRD [6,30]. CKD incidence was 15.5% among those without anemia, whereas 19.7% with anemia. The role of anemia in CKD development and progression is pivotal [6]. In addition to the well-known confounders of low GFR (<60 ml/min/1.73 m^2^), anemia was a significant risk attributable to low GFR. Besides gender, age and other attributable risks for low GFR were inexplicable by factors often measured; notably, proteinuria is the most potent factor among the measured variables for CKD progression prediction [6].

Anemia correction effect on CKD outcomes

According to a report published in 2023 from US data, severe anemia is prevalent and strongly linked to lower eGFR and various negative outcomes. Despite frequent testing for iron studies, low serum iron levels are widespread among those tested, and EPA treatment is often ineffective and not commonly utilized [31]. Clinical trials have shown the anemia correction benefit on renal outcome [9,32,33]. Research conducted on rats has indicated that using EPO to treat anemia could impact the development of kidney dysfunction. However, there is ongoing debate regarding EPO therapy on residual kidney function in humans. Kuriyama et al. stated that anemia exacerbates progression to ESRD and failure [33]. However, the study discovered that EPO can significantly decelerate CKD progression to ESRD, particularly in non-diabetic individuals. It is imperative to control BP, ensure appropriate Hct levels, and restrict dietary protein to achieve favorable outcomes [33]. Furthermore, Gouva et al. conducted a randomized trial that concluded that early commencement of EPO in non-severe anemia patients who are not yet undergoing dialysis significantly delays CKD progression and prolongs the time before RRT was required [32]. Another study conducted in 2004 by Mohanram et al. described that Hb is an independent factor for the progression of nephropathy to ESRD in type 2 DM [9]. Mild anemia (Hb < 13.8 g/dl) can increase the prospect of developing ESRD, especially in type 2 DM, as low Hb is a significant factor that can greatly contribute to the progression of DM-associated CKD to ESRD [9].

On the contrary, some reports and meta-analysis reports supported anemia correction but should not be complete (Hb < 13 g/dl) [6,34,35]. It was reported that the effect of a Hb level of 11.3 g/dl versus 13 g/dl related to increased CKD progression without incremental improvement in life quality [34]. Normalizing Hb in persons with type 2 DM who suffer from CKD was determined to be a safe procedure. However, it was found that this process does not effectively slow down the decline in kidney dysfunction and can lead to increased treatment costs [36]. Hb correction positively affects the LVH progression and decreases the CVD events, improving life quality [37,38].

Impact of anemia correction on CKD progression

Correction of anemia was linked to a considerable improvement in life quality [38]. ESA therapy protects the neuronal system and prevents kidney injury [39-41]. ESA attaches to receptors responsible for binding the tissue-protective receptor, specifically the EPOR/CD131 heterodimer, which protects tissues and regulates the immune system [39]. Moreover, it is worth noting that both parenchyma and immune cells can generate and release EPO when exposed to low oxygen levels. It is imperative to highlight that the EPO produced in situ exerts its effects in a paracrine-autocrine manner [39]. However, randomized clinical studies in ESRD and pre-ESRD stages demonstrated a preference for lower target Hb levels and no survival advantage from anemia correction. The lack of effect on survival may have unclear causes; however, it would only be supported if other CVD risk factors were professionally managed. HTN is often linked to ESA therapy, and dialysis-dependent patients are often poorly managed and are usually volume-overloaded, aggravating HTN and affecting HTN control [42,43]. It was reported that proteinuria in CKD improves after severe anemia correction due to inhibition of sympathetic nervous activity [44].

Numerous investigations conducted recently demonstrated that EPO has effects beyond erythropoiesis. Many tissues create EPO at the edges around damage sites in hypoxia or inflammation because EPO is essential for tissue protection and restoration [39]. These impacts were believed to be due to cell death inhibition and the blockade of inflammatory cytokines formation and action [45,46]. Research showed that EPO derivatives and EPO may also directly affect immunological cells, improving patients’ immunity.

Hypoxia

Hypoxia results from insufficient oxygen available at the tissue level, causing an imbalance in oxygen availability. This can occur due to decreased blood supply or reduced blood oxygen content (hypoxemia) [47]. The effects of hypoxia vary depending on the tissue; some can tolerate it for longer periods, while others, including kidneys, are severely damaged by shorter periods of low oxygen supply [48,49]. Multiple factors make the kidney more prone to hypoxia, including nephron blood supply pattern, the metabolic role of the kidneys, inflammatory reactions, apoptosis, poor angiogenesis, and macrophage accumulation, producing profibrotic cytokines like transforming growth factor β and activating renal fibroblasts [19,50].

Despite weighing less than 0.5% of our total weight, our kidneys receive 20% - 25% of cardiac output, making them the maximum perfused organs per gram of tissue. Surprisingly, despite this high blood flow (BF), the kidneys use 10%-20% of the oxygen delivered under normal conditions. Kidney hypoxia, particularly in the renal medulla, is extremely hazardous. Kidney hypoxia is a significant pathophysiological recognized character of acute kidney injury (AKI) and CKD; addressing this issue is crucial [51]. The partial oxygen pressure (pO2) levels in the cortex tissue typically range from 20 - 60 mmHg, while the outer and the inner medulla layers exhibit 15 - 30 mmHg and <15 mmHg, respectively [52]. These variations are directly attributed to the unique renal vascular structure of the medulla [53].

The intricacy of renal oxygenation physiology is a key hurdle to our comprehension [54]. Computational models may provide light on complicated physiological systems. However, these models must be based on correct anatomical and functional data, which are the realms of anatomists and physiologists. The current possible reasons that make the kidneys sensitive to hypoxia are still postulations. Understanding the kidney oxygenation physiology and generating innovative strategies to prevent AKI and CKD in the future might be possible. Our thoughts are based on several reasons and characteristics essential to explain the high sensitivity of the kidneys to hypoxia. These factors limit the oxygen amount renal tissue receives under normal circumstances or only become noticeable when renal oxygenation is compromised.

Patients with CKD develop chronic renal hypoxia due to different causes such as peritubular capillaries loss, tubulointerstitial fibrosis, downstream drop in tubulointerstitial BF caused by peritubular vessel blockages in injured glomeruli, Renin-angiotensin-aldosterone system (RAAS) activation, adrenaline, endothelin release, etc. [55,56]. All these lead to hypoxia due to vasoconstriction, poor perfusion, and oxygen stream to the kidney tissues. Moreover, chronic hypoxia induced by glomerular hyperfiltration leads to a discrepancy between the tubule's workload and the oxygen delivery rate in the earlier CKD stages. In addition, oxidative stress significantly contributes to increased oxygen demand [57]. Persistent hypoxia initiates inflammatory reactions, causing an excessive extracellular matrix buildup [57]. In addition to anemia, the kidney’s ability to receive enough oxygen decreases [58]. Interestingly, RAAS blockades are effective renoprotective drugs and individuals with CKD who have proteinuria respond to RAAS inhibition more favorably than those who do not [59]. According to many investigations, RAAS blockage improves tubulointerstitial hypoxia [60].

Moreover, obstructive sleep apnea (OSA)-induced hypoxia worsens renal disease via several detrimental systemic consequences, such as sympathetic activation, inflammation, and oxidative stress, intensifying the risk of nephron damage [61]. Besides the renal, vascular, and autoimmune diseases and lung complications, pulmonary involvement by these diseases causes hypoxia and hypoxemia (Figure 1).

Mechanisms of Renal Hypoxia

The kidneys are the body’s most perfused organs concerning their weight [62]. Oxygen concentrations in the renal cortex average 30 mmHg, while in the renal medulla are < 10 mmHg [63]. The significant difference in oxygen supplementation and oxygenation is due to how the kidney is structured and functions. Specifically, the pre-glomerular and post-glomerular arterial and venous blood supply in the kidney run parallel and are nearby over a long distance. The arranged anatomical layout pattern of arterioles and post-capillary venous structure enables oxygen to diffuse before reaching the capillary body. This arrangement makes BF for the medulla's internal and external layers lower than the cortex layers, affecting the delivered oxygen to the medulla [64,65].

**Figure 2:**
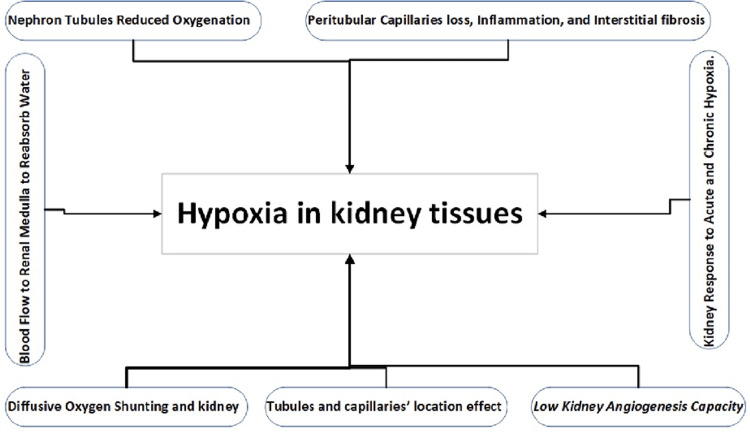
Possible mechanisms of renal hypoxia. The figure is the authors’ own creation.

The interlobular and afferent arteries form a sharp angle that impairs the capillaries supplying blood to the juxtamedullary glomeruli. This phenomenon is called plasma skimming, where red blood cells mainly flow to the cortex’s outer layer. In contrast, the juxtamedullary afferent arterioles collect the plasma and eventually flow into the vasa recta [19]. The tubule architecture and vasa recta arrangement pattern also limit oxygenation, making the kidney principally susceptible to environmental and physiological stresses that can cause ischemia due to the elevated oxygen demand in contrast to the impaired oxygen supply. The possible mechanisms of renal tissue damage due to hypoxia are illustrated in Figure 2.

Nephron tubules oxygenation

The normal kidney BF in a 70-kg-weighed individual is 1.25 l/min, corresponding to 700 ml/min of the kidneys’ plasma flow. Out of this 700 ml/min, around 140 ml/min are filtered by the glomeruli; 99% are reabsorbed [66]. The normal plasma sodium is 140 ± 5 mmol/l. Normally, 28 moles of sodium are filtered through daily, which equals 1.6 kg of sodium chloride. To keep the sodium plasma in balance, kidneys must reabsorb sodium. Sodium reabsorption by the nephron is mostly by active transport, requiring extra energy and oxygen. The active sodium reabsorption, which drives all other tubular transport activities, consumes roughly 80% of the delivered oxygen to the kidney in healthy circumstances [67]. These figures are only for sodium; the energy and oxygen consumption increase by including other substances like bicarbonate and glucose reabsorption, making the kidneys one of the highest oxygen consumers.

Another crucial consequence of sodium reabsorption dominance in determining the energy needs of the kidney is that renal oxygen consumption varies with the filtered load of sodium. Glomerulotubular balance describes the positive association between GFR and proximal convoluted tubule (PCT) sodium reabsorption (i.e., PCT sodium reabsorption load-dependence) [68]. Filtrate sodium load also affects sodium reabsorption in more distant nephron segments, resulting in GFR variations causing changes in tubular sodium reabsorption ability [69]. The GFR varies with renal blood flow (RBF) since it strongly relies on preglomerular vascular resistance [54]. Furthermore, autoregulatory systems that maintain the RBF and GFR in contrast to arterial pressure variations have developed [70]. These processes include the tubuloglomerular feedback and myogenic response from the macula densa, which work to balance the supply and demand of oxygen. However, their malfunction may relate to oxygen requirement and supply mismatch, causing renal tissue hypoxia. Furthermore, BF variations are frequently (but not always) associated with variations in the GFR, and hence the sodium-filtered load. Therefore, when the RBF and renal oxygen supply rise, renal oxygen consumption often increases accordingly [67]. Hence, the kidney's straightforward approach to prevent hypoxia is increasing RBF, which is far less successful in the kidney. Further, other functional imperatives have likely resulted in a poorly established hyperemic reaction to deoxygenated kidneys. Moreover, The PCT is an obligatory aerobic metabolizer nephron part [71]. Glycolytic capacity varies throughout the PCT, although poor [72]. Indeed, the PCT is a key location of glucose reabsorption and synthesis, which cannot occur effectively unless glycolysis (both processes require oxygen) also occurs. Thus, the PCTs synthesize ATP from substrates other than glucose and need oxygen (oxidative metabolism) during hypoxia because they have few backup plans. Moreover, the PCT can only sustain its transport function via oxidative metabolism and is inhibited by hypoxia [73]. Additionally, tubular components of the renal outer medulla, particularly the thick ascending limbs of the loop of Henle (TALH), may perform glycolysis and generate ATP without oxygen [72]. It is worth noting that the physical location of outer medullary TALH makes their functions even more risky.

Peritubular capillaries loss, inflammation, and interstitial fibrosis

Capillary rarefaction has recently been labeled as one feature of CKD in various etiologies [74]. The processes behind microvasculature loss in CKD are complicated and far from understood [75]. Nonetheless, they provide a possible therapeutic target for delaying the CKD course [75]. Interstitial fibrosis and hypoxia have been found to have roles [75]. Fine and colleagues postulated around 20 years ago in their “chronic hypoxia hypothesis” that CKD may be attributed to a vicious cycle of tubular injury, inflammation, interstitial fibrosis, and capillary loss.

One critical claim concerning the function of capillary rarefaction in renal hypoxia development in CKD is how sensitive cortical tissue oxygenation is to capillary loss. When assessed using unbiased stereological techniques, the surface area density of peritubular capillaries in the kidney (239.7 cm^2^/cm^3^) is equivalent to that of other densely perfused internal organs such as the brain, liver, and lung [76]. Similarly, erythrocyte speed in peritubular vessels is 1 mm/s, which is close to that recorded in the brain (0.8 mm/s) [77-78]. Therefore, there is no obvious explanation for why the kidney would be more prone to the impacts of capillary rarefaction despite the similar erythrocyte flow speed. However, organs such as the brain and skeletal muscle can recruit additional capillaries when exposed to hypoxia or when metabolic needs increase [79,80]. In contrast, kidneys appear less capable of accommodating these changes by recruiting new capillaries [81].

In a computer model of oxygen transport in the cortex, investigating the relative relevance of the many parameters that impact renal cortical oxygenation [82]. Cortical layers of PO2 in the model were sensitive to the alterations in RBF, GFR (and hence sodium’s filtered load), and oxygen usage efficiency for sodium reabsorption, as anticipated from basic principles. The model simulations further showed that cortical PO2 is extremely sensitive to changes (especially reductions) in peritubular capillary surface area, suggesting that capillary rarefaction is vital in making the kidney hypoxic in CKD. One notable finding from the model simulations is that the surface area of the peritubular capillaries’ reduction can increase the sensitivity of cortical tissue PO2 to various challenges that affect oxygenation, as in decreased RBF, increased sodium filtered load, or decreased efficiency in using oxygen for sodium reabsorption. This could help explain why people with preexisting CKD are more susceptible to developing AKI [83].

Low kidney angiogenesis capacity

The kidney’s endothelial cells have a low proliferation ability, contributing to a quick transition from AKI to CKD [84,85]. The poor proliferative capacity of renal endothelial cells could be due to at least their relative insensitivity to pro-angiogenic factors, including vascular endothelial growth factor (VEGF-A), which could be due to the influence of unidentified negative regulatory signals. The kidney controls Hct by releasing EPO, stimulating erythrocyte synthesis, and controlling salt and water excretion and plasma volume. In reaction to hypoxia or anemia, renal hypoxia raises EPO synthesis and circulating levels, positively driving erythropoiesis. Hypoxia also promotes hemoconcentration by abruptly stimulating diuresis [86-88]. Hypoxia would interrupt crucial activities if it encouraged vessel genesis in the kidney. Thus, the low proliferating capacity of kidney endothelial cells may be an evolutionary tradeoff that preserves the kidney’s critmeter function [89]. This might result in greater kidney sensitivity to capillary rarefaction, resulting in hypoxia and renal disease.

Kidney response to acute and chronic hypoxia

Most organs have well-developed systems for increasing oxygen delivery in reaction to hypoxia by local mechanisms, such as increasing overall BF and recruiting additional capillaries (known as “functional” or “active” hyperemia), which are driven by local hypoxia [79]. Following kidney ischemia, reactive hyperemia due to the release of local vasodilators may occur [90]. When animals under anesthesia are given hypoxic gas mixtures that result in lower arterial PO2, BF rises in certain organs, such as skeletal muscle, but not to a similar extent in the kidneys [56,91]. This may seem counterintuitive unless we examine the kidney's critical involvement in controlling blood volume's cellular and noncellular components. As stated earlier, the kidney is the body’s “critmeter” because it synthesizes EPO and stimulates erythropoiesis [89]. Renal hypoxia triggers EPO production [86,87]. Furthermore, the kidney adjusts extracellular and plasma volumes via its function in fluid and electrolyte excretion [92]. Changes in neurohumoral response mechanisms and arterial pressure work to modify renal hemodynamics and tubular function as a sequence to variations in extracellular fluid and blood volume [93]. Local BF might intervene with these processes if the kidney manifests hypoxia by fluctuating renal vasculature tone. The kidney's incapacity to safeguard itself from acute hypoxia by rising BF is probably a result of evolutionary pressures prioritizing the maintenance of other renal functions. Preserving extracellular volume and blood oxygen-carrying capacity may take precedence over shielding the kidney from hypoxia.

Chronic hypoxia initiates pathological changes in tubular endothelial cells, including apoptosis, inhibiting redifferentiation following regeneration, and conversion to myofibroblasts [94,95]. Hypoxia also induces monocytes to express the 2-integrin of adhesion molecules, intracellular adhesion molecule 1, and monocyte chemoattractant C-C motif ligand 2 and C-X3-C motif ligand 1 [96]. Hypoxia can cause changes in gene expression through various mechanisms, including chromatin-remodeling, histone modification, HIF-1 factor transcription, and alpha-binding protein rich in purine [97,98]. Hypoxia causes macrophage migration into the kidneys, promoting pro-inflammatory adhesion molecules and chemokines formation, producing profibrotic cytokines like transforming growth factors and activating renal fibroblasts [99,100]. Furthermore, hypoxia directly activates fibroblasts, strengthening extracellular matrix materials deposition due to increased collagen formation and synthesis of tissue-metalloproteinase-I inhibitor while diminishing collagenase expression [101]. Fibroblast activation, inflammatory cell recruitment, and tubule cell injury contribute to tubulointerstitial fibrosis development. The formed fibrosis exacerbates hypoxia by widening the diffusion space between capillaries and the tubule epithelial cells, thereby dipping oxygen diffusion effectiveness [102]. Consequently, tubulointerstitial fibrosis and hypoxia constitute a vicious pathologic cycle that causes CK progression [63]. The problem is worsened by gene changes that cause low oxygenation and activate histones. This leads to more endothelin 1 production, constricting blood vessels and lessening kidney oxygen flow [103].

Diffusive oxygen shunting and kidney

The renal cortex has arteries and veins arranged in a countercurrent pattern. The veins partially surround the arteries, creating a path for oxygen diffusion [63,103]. The arterial blood in the kidneys carries oxygen, but because of the arteriovenous (AV) oxygen shunting, some oxygen is redirected to nearby veins instead of reaching the renal glomeruli and tubule tissue. The direct shunting impact on the kidney's sensitivity to hypoxia has not been measured but estimated through computer models. It is crucial to assess the importance of this phenomenon. The models were formulated with varying assumptions and produced different results, so more research is needed.

Gardiner and colleagues’ first one-dimensional model estimated that 13% of the oxygen conveyed to the kidney via the renal artery is diverted to the renal veins [104]. The model was founded on two key assumptions. Nordsletten et al. used a model depending on a dataset of branching arteries and veins sizes and lengths in the cortex [105]. In addition, another team model was “calibrated” using a series of oxygen tension measurements in structures on the cortex surface [106]. The same study revealed a 7 mmHg differential in PO2 between the efferent arteriole and the renal vein [106]. The segment-wise model indicated that AV oxygen shunting accounts for just 1% of the total renal oxygen supply (i.e., > 10-fold fewer than the reported values) [107]. Both models were criticized because they needed to account for differences in the spatial organization of veins and arteries throughout the length of renal circulation [108,109]. Olgac and Kurtcuoglu and Lee and colleagues independently developed models incorporating realistic information about axial and radial geometries of renal preglomerular circulation [110,111]. In the basic scenario of “physiological conditions,” both models anticipated AV oxygen shunting of 1% of the renal oxygen supply, supporting the previous reports.

The most reliable data shows that renal AV oxygen shunting is not a major concern, but it still has an impact under physiological conditions. Specifically, simulations used by Lee and colleagues’ model indicate that it can lower the PO2 of blood in the glomerular capillaries by 6.6 mmHg, even in normal circumstances [111]. It is remarkably important in cases of renal ischemia, where the amount of oxygen being shunted is expected to increase. On the contrary, the overall oxygen flow through the arterial circulation decreases [111]. More research into these models could help us understand the physiological and pathological relevance of renal AV oxygen shunting [112].

Oxygen diffuses from the long descending to ascending vasa recta vessels in the renal medullary circulation [113]. As with AV oxygen shunting in the cortex, experimental physiologists' efforts and computational models have significantly contributed to assessing blood shunt in the medullary part of the nephron. Simulation models showed that oxygen shunts between vasa recta vasculatures are physiologically substantial and a key contributor to making the inner medulla sensitive and more vulnerable to hypoxia [114]. Published data estimated that 3% of the oxygen in the descending vasa recta is shunted by diffusion to the neighboring ascending vasa recta under standard physiological circumstances [114].

The selection forces resulted in the profound countercurrent arrangement of arteries and veins in the cortex and the descending and ascending vessels of the vasa recta in the medulla, allowing diffusive oxygen shunting. The countercurrent configuration of the vasa recta in the medulla is crucial to medullary hyperosmolality, which is vital for urine-concentration processes [115]. As a result, this arrangement is most likely the result of a terrestrial environment [113]. The processes that cause the exceptionally close interactions between internal kidney arteries and veins are yet unknown. According to O'Connor and Evans, it might be due to the structural antioxidant defense system that protects the kidney from hyperoxia and oxidative stress [116]. However, this is implausible unless the present AV oxygen shunting size estimates are significantly underestimated. It is also possible that this unusual anatomy evolved because of selection pressure for the countercurrent exchange of molecules other than oxygen (e.g., carbon dioxide, NO, hydrogen disulfide, or ammonia); however, it appears unlikely [113].

Blood flow to renal medulla effect on solutes and fluid reabsorption

It is also commonly believed that the countercurrent layout of ascending and descending vasa recta facilitates the medullary peritubular osmolarity gradient’s development, by water reabsorption, urea cycle, selective permeability to water and sodium, and other mechanisms by nephron tubules. Furthermore, maintaining this solute gradient requires modest medulla BF [117]. This is because the efficacy of a countercurrent system decreases when transit time decreases, as would occur if medulla BF increases. Thus, the terrestrial environment must have exerted significant selection pressure for a modest medullary BF, increasing the renal medulla's sensitivity to hypoxia, and increasing kidney damage.

Tubules and Capillaries’ Location Effect

Anatomically, the renal medulla’s vascular blood supply architecture differs from the cortex [118,119]. Computational model simulations show that this design significantly impacts the radial gradients in oxygen tension inside and around the vascular bundle and oxygen dispersal into the medulla [52]. The core part of the vascular bundles of the outer medulla's inner stripe comprises descending vasa recta that terminates in the inner medulla [118]. The TALH is located on the perimeter of the vascular bundles; the relatively longer distance from the descending vasa recta ends might restrict their oxygen supply [120]. The adaptive advantage of this topography has yet to be discovered. It is claimed that this might result in osmotic gradient lateralization, promoting sodium and water reabsorption, and increasing oxygen demand [118,119]. These issues need further exploration and studying.

Computational models predict that the TALH’s position at the periphery of the vascular bundles in the inner stripe, where oxygen supply is primarily from ascending vasa recta, combined with their high metabolic demand, makes this tubular component particularly susceptible to hypoxia [120]. This theory is reliable with the widespread discovery of medullary TALH damage in AKI and fits well with the commonly known causes of AKI [121]. Sgouralis and colleagues demonstrated severe TALH damage due to hypoxia associated with AKI following cardiopulmonary bypass in rats model [122]. According to Corredor et al., the region between the bundles in the lower outer medulla inner stripe (where TALH is located) experiences a lack of oxygen during major surgeries like cardiopulmonary procedures [123]. This supports the findings of animal studies. This may clarify why about a quarter of patients who undergo these surgeries develop AKI, which typically has negative consequences [123,124].

Hypoxia effect on CKD onset and progression

Tissue hypoxia is a well-known factor that affects tissues and cell death. Hypoxia is precipitated by varied factors, such as local oxygen availability, the energy needs of cells, and their natural tolerance to hypoxia [125]. Furthermore, anemia is a considerable physiological cause of tissue hypoxia due to the decreased ability of Hb to deliver enough oxygen to tissues (anemic hypoxia). For a long time, hypoxia was considered a cause of tubulointerstitial damage. It was reported that hypoxia is an integral pathophysiological feature of CKD. Additionally, hypoxia is meticulously linked to renal inflammation, fibrosis, anemia, and tissue damage [125].

Hypoxia causes of CKD development are discussed in different reports [19,60,125]. Advancements in understanding oxygen’s effect on erythropoiesis regulation led to new therapies for anemia-related CKD. Hypoxia-inducible factor (HIF) -1 and 2 control EPO production and iron metabolism as a response to low oxygen levels. Activation of the HIF axis could help treat renal anemia by stimulating the body’s response to low oxygen levels, preventing hypoxia. Furthermore, it was observed that anemia impairs hypoxia responses to therapy [126]. Hence, anemia correction is vital to prevent both kidneys’ hypoxia; however, the correction must be within the recommended Hb standards.

Studies highlight chronic hypoxia’s role in tubular interstitium damage, leading to ESRD. When hypoxia is severe, tubulointerstitial injury links with peritubular capillaries loss. Furthermore, interstitial fibrosis reduces oxygen diffusion, increasing ischemia to interstitium and tubule cells. Hypoxia of tubule cells promotes epithelial-mesenchymal trans-differentiation and cell apoptosis, intensifying further kidney tissue fibrosis and chronic hypoxia worsening. Several factors were reported leading to tubulointerstitial hypoxia during the initial stages of kidney damage [60]. Mismatch in vasoactive chemicals, glomerular damage, and efferent arteries vasoconstriction reduce BF in the capillaries around the glomeruli and induce their damage [127]. Angiotensin II narrows efferent arterioles and causes oxidative stress, making it hard for tubular cells to utilize oxygen effectively [60,125 ,127]. Therefore, understanding and using these mechanisms to prevent hypoxia is important, and it might be beneficial to invent new therapies. Hence, further investigation of this assumption is required.

Inherent cellular resistance to hypoxia, cellular energy needs, and local tissue oxygen tension influence hypoxia development. The nephron’s PCT cells are principally vulnerable to hypoxic injury, and tubular cell extent damage predicts kidney disease outcome [125,128]. Renal fibrosis starts with pericyte differentiation into active myofibroblasts in the interstitium layer due to hypoxia [129]. Moreover, hypoxia causes peritubular endothelial activation, leukocyte stasis, and a reduction of peritubular capillaries BF, contributing to capillary architecture loss, hypoxia aggravation, more nephron death, and interstitium fibrosis [130]. These issues are not well studied, and their therapeutic benefits are not well explored. Therefore, new study projects are needed.

Oxygen tension variation in the kidney cortex and the tubulointerstitial tissue significantly depends on post-glomerular capillary BF for oxygen delivery. Thus, microcirculation damage from an upstream blockage may instantly lower the tubulointerstitial oxygen tension. Additionally, several variables alter tissue cell sensitivity to hypoxia, including tissue metabolic rate and HIF pathway activation. It is compiled and explores physiological variables that could make the kidney vulnerable to hypoxia [52]. Understanding these variables might help in new therapeutic and preventive approaches (Figure 2).

Overweight and Obesity

The rate of overweight and obesity is rising globally, negatively affecting body function, including kidney, manifesting with the eGFR decline [131-133]. Morbid obesity leads to hypoxia, hypoventilation syndrome, and OSA. All these consequences of obesity induce hypoxia and hypoxia, especially to highly-demand organs such as kidneys. A study of middle-aged individuals found a link between fat body content and proteinuria [134]. Proteinuria and focal segmental glomerulosclerosis occur frequently in obese individuals [135,136]. Interestingly, proteinuria improves with weight loss [136,137]. In overweight and obese, in addition to proteinuria, systolic HTN, and hyperuricemia were strong markers of reduced kidney function [138,139]. The cause of CKD onset and progression in overweight and obese individuals is hypoventilation. OSA is repeatedly found in people who are overweight [140]. In OSA, CKD and CVD rate is high, indicating a strong link between these conditions [6,141]. Authors claimed that OSA happens in 50 - 60% of CKD patients developed ESRD [142]. Others reported that the OSA prevalence rate is 10-fold higher in CKD individuals than in the general population [143].

The influence of fat body content and metabolic damage on CKD might differ for people of different races [133,144]. Lew et al. conveyed that obese individuals with low lean mass were more expected to have proteinuria; furthermore, they demonstrated the link between body mass index and proteinuria, which differs for different races. Hence, this racial difference needs further studying [145].

Hyperglycemia

Diabetic nephropathy is allied to reduced kidney oxygen tension [146]. Multiple mechanisms have been connected to this phenomenon’s occurrence. Hyperglycemia causes renal mitochondria, nicotinamide uncoupled NO synthase, and adenine dinucleotide phosphate oxidase to form reactive oxygen species (ROS) [147]. The ROS (superoxide) interacts directly with NO, decreasing peroxynitrite production and NO bioavailability. L-arginine is diminished by an independent process of ROS, further decreasing NO's bioavailability [146]. Independently, decreased NO and increased ROS increase oxygen utilization [146]. In addition, decreased NO causes vasoconstriction, decreasing kidney perfusion, and less oxygen delivery [148]. Hyperglycemia also reduces kidney perfusion by reducing glomerular arterioles diameter by encouraging extracellular collagen deposition in vascular smooth muscle, mesangial proliferation, and distal convoluted tubule epithelium cells [149].

Hyperglycemia causes sustained protein kinase C activation and nuclear factor kappa-light-chain-enhancer of activated B cells, stimulating osteopontin synthesis [149,150]. The osteopontin activates its 3-integrin receptor, which sends signals to encourage collagen synthesis and other proteins [149]. Along with eliciting protein kinase C, hyperglycemia increases intercellular adhesion molecule 1 expression by mesangial cells, thereby promoting glomerular injury caused by mononuclear cell infiltration [150]. Interestingly, analysis of diabetic nephropathy in obese mice supported the link between kidney tissue hypoxia, hyperglycemia, and CKD development [151]. Furthermore, hyperglycemia, increased ROS synthesis, capillary loss, arteriolar vasoconstriction, and a drop in quiescent and maximal BF increase the hypoxia risk [151,152].

Hypertension

In systemic HTN, multiple vasoconstrictors such as RAAS activation products, endothelin, and constrictor prostaglandins formation occur [153,154]. BF decreases in the constricted blood vessels, declining end-organs oxygen delivery, including the kidneys [155,156]. In addition, HTN causes increased kidney metabolic activity, upsurging oxygen consumption twice compared to oxygen in non-hypertensive for handling the same sodium amount [157]. In studying hypertensive rodents, kidney medulla, and cortex, oxygen tension was almost 10 mmHg lower than in non-hypertensive rodents and hypertensive patients [158-160]. The first documented evidence of HTN impact on CKD progression was dated to 1914 [161]. Since then, HTN has been a recognized hypoxia-inducer risk factor for renal failure [162]. HTN also promotes kidneys to produce ROS such as hydrogen peroxide, peroxynitrite, superoxide, and hydroxyl radicals, aggravating hypoxia, and its negative effects. Furthermore, the raised intrarenal angiotensin II promotes ROS synthesis and binding to its type 1 receptor, which then transduces signals to stimulate the pro-oxidant enzyme nicotinamide adenine dinucleotide phosphate oxidase [163]. The antioxidant enzymes superoxide dismutase 1 and 3 and isoforms of NO synthase expressions are reduced in HTN, thereby increasing ROS production [163], which causes more renal hypoxia. Improving HTN control by angiotensin receptor antagonists, antioxidants, and angiotensin-converting enzyme inhibitors recovers kidney oxygenation and kidney dysfunction in hypertensive rodents [158,164]. Therefore, HTN control is critical to prevent kidney hypoxia and CKD early onset and progression.

Hypercholesterolemia

Studies have unequivocally established a link between raised cholesterol levels, impaired kidney oxygenation, and heightened susceptibility to kidney ischemia [165]. Mechanisms are driven by cholesterol's role in defining the cell surface physicochemical properties. The erythrocyte membrane consists of cholesterol, making it non-polar and hydrophobic. The extraordinary cholesterol erythrocyte membrane content declines its flexibility and fluidity, impairing the erythrocyte's abilities to absorb and dispense oxygen to the kidneys. Furthermore, hypercholesterolemia increases cholesterol deposition in kidney structures [166], compromising diffusional oxygen delivery [167]. Moreover, cholesterol metabolism may have a considerable influence on renal hypoxia risk. The research found that esterified cholesterol formation and cholesterol transport protein translocator protein formation are linked to resistance against kidney injury caused by hypoxia [168]. However, sustained hypercholesterolemia impedes molecule expression, compromising cells' protection mechanisms during ischemia [169,170].

Atherosclerosis

Multiple interactions between detrimental incentives and reparative responses or arterial wall's restorative lead to atherosclerosis development [171]. Following endothelial damage, direct cell-cell interaction, growth factors, and chemotactic secretion induce the enrollment of monocytes to subintimal layers, smooth muscle proliferation, and increase matrix protein synthesis. After monocyte recruitment, they become macrophages and gather lipids, eventually forming foam cells. This process, along with the presence of T-lymphocytes, forms the fatty streak, an early indicator of atherosclerosis in histopathology.

Atherosclerotic lesion progression is characterized by macrophage-lipid-laden accumulation. In early atherosclerotic renal artery stenosis phases, compensatory alterations in arteriovenous shunting, GFR, and tubular sodium reabsorption decrease oxygen consumption or raise oxygen availability. Direct intrarenal oxygenation measurements revealed regional hypoxia in the cortex and the medulla [172,173]. The intrarenal hypoxia pattern heterogeneity is mostly due to intrinsic distinctions in the kidney medulla and cortex vasculature physiology [174]. Because of these disparities, vascular stenosis induces localized minute vasculature dysfunction, inducing cortical hypoxia, leading to RAAS activation, kidney malfunction, venule and arteriole rarefaction, fibrosis, kidney atrophy, and ESRD due to ischemic nephropathy [175-177].

Preserving the microvascular structure by intrarenal administration of VEGF-A diminishes renal fibrosis. VEGF-A preserves renal hemodynamics and improves kidney dysfunction in the induced severe renal artery stenosis model, highlighting the link between atherosclerosis-induced kidney hypoxia and CKD [175]. Additionally, the renal disease frequency is markedly reduced when femoropopliteal angioplasty is conducted to revascularize in widespread atherosclerosis cases [178]. Furthermore, different clinical studies have verified the efficiency of renal angioplasty and renal artery stenosis stenting in improving renal dysfunction [179,180]. Whether reoxygenation of the kidneys through this surgical procedure is preferable to that tempted by medical treatment is debatable. However, both strategies are designed to treat renal hypoxia. The practice guidelines of the American Heart Association and the American College of Cardiology Foundation outlined the importance of hemodynamically substantial renal artery stenosis management for improving kidney oxygenation [181].

Cigarette consumption and environmental pollution

Active and passive smoking are independent factors for the de novo CKD onset in otherwise healthy subjects [182,183]. Heavy continual cigarette smoke is alleged to contain a variety of harmful constituents, such as cadmium [184,185]. A direct correlation exists between the amount of cigarettes smoked and renal dysfunction intensity and the likelihood of CKD onset or kidney transplant failure [185,186]. Additionally, smoking cessation declines the developing CKD risk and benefits patients who already have CKD [182,187].

Tar accumulation in the lungs produces a physical barrier, which inhibits alveolar gas exchange, producing systemic hypoxia due to less oxygen exchange at the alveolar level. Moreover, tar reduces erythrocytes' oxygen availability by encouraging inflammatory responses that necessitate increased oxygen utilization [188]. Carbon monoxide in cigarettes smoked binds reversibly to various body heme proteins (Hb, cytochrome P450, myoglobin, and cytochrome oxidase proteins). Dissociation of the carboxyhemoglobin bond is less than the oxyhemoglobin bond, significantly impairing oxygen transport in severe cases [189,190].

Moreover, nicotine promotes NO breakdown and activates parasympathetic nerves. Furthermore, nicotine binds various nicotinic acetylcholine receptors on mesangial, vascular smooth muscle, endothelial, and proximal and distal convoluted tubule cells [189,190]. In addition, nicotine activates protein kinases, enhancing ROS production by mesangial cell proliferation and extracellular milieu deposition via pathways involving upraised expression of cyclooxygenase 2 and increased extracellular signal-regulated protein kinases 1 and 2 phosphorylation, c-Jun N-terminal kinases, protein kinase, and activator protein 1B [189,191,192]. This abnormal multiplication and deposition of nicotine promote hypoxia-like hyperglycemia by promoting oxygen-consuming inflammation. Increased unphosphorylated signal transducer and activator of transcription 3 expressions cause PCT cells to produce the pro-inflammatory cytokines transforming growth factor 1 and monocyte chemotactic proteins. In addition, HTN risk increases significantly with the daily smoked cigarette number and smoking duration, augmenting kidney hypoxia and its effects [188]. Therefore, it is reasonable to infer that smoking is a principal risk factor for CKD development.

Passive inhalation of carbon monoxide from secondhand smoke or air pollution induces hypoxic effects comparable to active cigarette smoking [193]. Sulfur dioxide and nitrogen dioxide can convert Hb into methemoglobin, rendering it ineffective for oxygen transport [194]. Lead and arsine are central nervous system toxins that can impair respiration, causing hypoxia. These gases and substances are the prevalent pollutants in vehicle exhaust emissions. Ozone induces acute arterial constriction, restricting BF and oxygen delivery [195]. Ultrafine particulates with an aerodynamic are released by diesel engines, entering the bloodstream directly, restricting oxygen delivery via vasoconstriction, incrementing blood viscosity and vascular inflammation risk [195,196].

Fine particles released by diesel usage accumulate in the pulmonary alveoli, creating an inflammatory response by stimulating the local synthesis of inflammatory cytokines (interleukin 6, tumor necrosis factor) and vasoconstrictor endothelin 1 by the activated alveolar cells and macrophages [196,197]. Like diesel particulates and cigarette tar, wheat, flax, coal, silica, rice, cotton, silk dust, fiberglass, and asbestos fibers can accumulate in the airways, restricting gas exchange and eliciting oxygen-consuming inflammation [198,199]. Agricultural dust such as cotton, rice, flax, wheat, and wood are laden with lipopolysaccharide Gram-negative bacterial endotoxins that cause chronic airway inflammation, resulting in chronic lung dysfunction and hypoxia [199].

Air pollution is reported to impact kidney oxygenation significantly. This can be due to inflammation consuming oxygen, reducing the lungs’ capability to exchange gases. Studies demonstrated that exposure to amosite asbestos or passive smoking can lead to glomerulosclerosis and tubulointerstitial fibrosis development in rodents. Furthermore, silica, fiberglass, or solvent exposure is linked to kidney malfunction [19]. All these issues require further research and exploration.

Obstructive respiratory sleep disorders

In CKD, sleep disorder prevalence is significantly greater than the 20% observed in the general population [200]. Central SA is characterized by a protracted absence of drive to breathe, whereas an aberrant pharyngeal airway collapse characterizes OSA. Between the ages of 30 - 65, approximately 5% of females and 16% of men are afflicted with SA. According to longitudinal cohort studies of the interrelation between SA and renal dysfunction, SA is the independent risk that accelerates kidney function decline [201-203]. These observations led to the conclusion that both SA and CKD pose a risk to each other [141]. SA may be produced by autonomic nerve injury caused by uremic neuropathy, affecting pharyngeal narrowing, abnormal baroreceptor activity due to excess fluid, and elevated uremic toxins levels [141].

SA induces CKD via multiple mechanisms that encourage renal hypoxia [204]. Apnea causes inadequate ventilation, compromising gas exchange [205,206]. Long-term exposure to repetitive episodes of hypoxia-reoxygenation activates nicotinamide adenine dinucleotide phosphate-oxidase 2, producing ROS [207,208]. The tissue injury caused by these ROS is aggravated by alternating hypoxia, reducing antioxidant expression in the kidney [209]. Moreover, intermittent hypoxia galvanizes the renal RAAS and stimulates the sympathetic system, leading to vasoconstriction and increasing vascular resistance [210,211]. These processes work in concert to produce ischemia and generate renal fibrosis, HTN, and poor renal oxygenation [212,213].

Nocturnal hypoxia alters the heart rate parasympathetic control and induces LVH, inducing further impairment of oxygenation [214]. Furthermore, SA activates nuclear factor B, initiating an events cascade that increases tumor necrosis factor production, interleukins (6, 8, 18), C-C motif, and C-reactive protein [215,216]. Moreover, systemic inflammation and apnea-induced ROS, HTN, and platelet aggregability promote atherosclerosis progression and its mechanisms for renal hypoxia development. Research has demonstrated that chronic intermittent hypoxia and a high-fat diet can lead to atherosclerotic plaque development. Continuous positive airway therapy in SA significantly improves renal oxygenation, which could significantly decline CKD onset and progression in these individuals.

Environmental, pathophysiological, and behavioral interactions and hypoxia

Environmental, pathophysiological, and behavioral risk factors distinctly decrease systemic or kidney-specific oxygenation. Renal cells respond with protecting mechanisms centered on extracellular signal-regulated kinases 1 and 2 phosphorylation and HIF-1 and -2 stabilization [101,217-219]. These defensive mechanisms lead to pro-angiogenic (isoform 164 of VEGF-A) factors upregulation, protecting from capillary rarefaction and matrix metalloproteinases stimulation, improving matrix repair, and preventing fibrosis [220,221]. Additionally, short exposure to hypoxia increases antioxidant (nuclear factor erythroid 2-related factor 2, metallothionein I, and heme-oxygenase 1) kidney expression, which protects from fibrosis and inflammation caused by ROS [209,222,223].

Prolonged hypoxia increases natural antisense HIF-1, causing a decline in HIF-1 expression by making its mRNA unstable [224]. The increased ROSs also target the HIF-1 protein degradation via the ubiquitin-proteasome system [225,226]. Hypoxia alters HIF-2 expression, leading to an alteration in the quantitative nature of HIF far from subunit 1 and towards subunit 2 [224]. The kidney’s protective mechanisms fail to activate or downregulate under prolonged hypoxia due to the change in HIFs expression [209,219]. Therefore, the pro-angiogenic factors, such as VEGF-A isoform 164 expressions, are repressed, whereas dysangiogenic factors (VEGF-A isoforms 120 and 188) expressions are amplified, resulting in capillary rarefaction [227,228]. Reduced metalloproteinases and augmentation of their inhibitors expression and extracellular milieu proteins contribute to high fibrosis risk [101]. Antioxidant expression is either not induced or is declined, allowing increased ROS expression that positively promotes tubulointerstitial fibrosis via necrosis, apoptosis, epithelial-mesenchymal transition, interstitial fibroblast activation, endothelial cell proliferation, and pericytes transformation [17,209,229,230].

Additionally, protracted hypoxia induces nephron endothelium to synthesize pro-inflammatory adhesion molecules, chemokines, and cytokines, which upgrade macrophages already existing in the kidney and recruit further inflammatory cells out of the circulation [231]. Activated macrophages aggravate an inflammatory response by additional cytokines production (tumor necrosis factor 1 and interleukins). Moreover, activated macrophages encourage fibrosis by generating pro-fibrotic cytokines (transforming growth factor 1) [232,233]. Inflammatory cell and fibroblast activation combined with endothelial proliferation, apoptosis, and epithelial-mesenchymal transition to generate tubulointerstitial fibrosis following protracted hypoxia, reducing oxygen and blood perfusion. Furthermore, prolonged hypoxia excites the cortex to upregulate endothelin 1 and its type A receptor, potent vasoconstrictors, suffocating it, precipitating CKD development [102,234] .

Increased intercellular adhesion due to cell damage increases inflammatory cell recruitment into hypoxic tissues [235]. The intercellular adhesion molecules on renal tissue cell surfaces are essential adhesive molecules [236]. On the recruited activated leukocyte surface, the antiadhesion molecules (CD43 and CD45 and the 2-integrin group of proadhesion molecules) are particularly important [237,238]. Once leukocytes are activated, CD43 and CD45 are down-regulated along with their sialylation, and the pro-adhesive 2-integrin family is simultaneously up-regulated [239,240]. Leukocytes acquire adhesive phenotype agents that can extravasate and infiltrate into the kidneys for inducting this reciprocal manifestation of proadhesion and antiadhesion factors effects.

Recurrent AKI episodes

AKI is reversible if diagnosed early and promptly treated [241,242]. The preponderance of AKI leading causes results in hypoxic kidney conditions. These comprise sepsis, which causes renal vasoconstriction via endothelin release. Radiocontrast studying increases oxygen utilization for electrolytes and other reabsorption and decreases local inner medullary region BF [243,244]. AKI is also commonly caused by a more widespread diminution in BF. In decompensated liver cirrhosis, massive hemorrhage, treatment with nonsteroidal anti-inflammatory medications, CHF, massive renal artery stenosis or closure, and reduced RBF [241]. In addition, surgical procedures in which BF is accidentally or intentionally decreased bear an inherent risk of AKI [245,246]. Moreover, massive blood loss frequently occurs during and after large hepatic resections, resulting in unintended BF reductions during or after surgery [245]. Clamping main blood vessels during organ transplantation, large aneurysm, and cardiopulmonary bypass surgery reduces RBF, leading to ischemia and hypoxic damage of the kidneys [247]. A meta-analysis, clinical follow-up studies, and epidemiological have all demonstrated a strong association between recurrent AKI attacks and CKD onset [248-250]. This link is demonstrated even in patients whose renal function has returned to normal after AKI [251]. AKI severity and AKI attack recurrence rates are remarks for CKD development [252]. These conclusions have established a causal link between CKD onset and AKI [253]. Cisplatin, contrast agents, rhabdomyolysis, and oxygen tensions < 10 mmHg initiate hypoxia, precipitating AKI by inducing ischemia [96,254].

In humans, impaired kidney oxygenation was experienced in AKI [255]. It is imperative to understand that kidney structure hypoxia can persist for up to five weeks after the recovery stage and not just during the early AKI stage [96,256]. The continued hypoxia triggers the kidney to downregulate the pro-angiogenic isoform 164 of VEGF-A, upregulating the dysangiogenic isoforms [257-258]. Accordingly, the kidney vascular architecture is not maintained, as the capillary number, individual capillary diameter, and area decrease, which consequently causes capillary rarefaction, diminishing oxygen delivery [257-258]. Chronic hypoxia causes several pathological changes inside tubule epithelial cells. These include apoptosis, redifferentiation and regeneration prevention, and myofibroblast transformation [95,259]. Additionally, hypoxia enhances monocytes to present the 2 integrin adhesion molecules group, intracellular adhesion molecule 1, and the monocyte chemoattractants (C-C motif ligand 2 and C-X3-C motif [19,97,258,260,261]. On the other hand, chromatin-remodeling, histone modification, and the transcription factors (HIF-1 and purine-rich binding protein alpha) mediate these hypoxia-dependent alterations in gene expression [19,260]. Hypoxia promotes the macrophages’ disposition to the kidney, which produces profibrotic cytokines (as a transforming growth factor) and activates renal fibroblasts by inducing pro-inflammatory adhesion molecules and chemokines [99,262]. Hypoxia also directly activates fibroblasts to increase extracellular matrix deposition due to increasing collagen and tissue-metalloproteinase-I production inhibitors while diminishing collagenase expression [101,263]. The activated fibroblasts, inflammatory cell enrollment, and injury to tubule epithelial cells contribute to tubulointerstitial fibrosis development. Hypoxia and tubule interstitial fibrosis constitute a pathological cycle that precipitates CKD progression [200]. This cycle is aggravated by modified hypoxia-inducing gene-activating histones that stimulate endothelin 1 expression, thereby decreasing oxygen transport to the kidneys. Hypothetically, modulation or interruption of these processes could reduce hypoxia and prevent AKI and CKD.

Prevention of CKD onset and progression in relation to hypoxia

Therapeutic strategies targeting prolonged hypoxia can prevent different varieties of kidney illnesses. Strategies include using EPO or its derivates to treat anemia and blocking the RAAS blockade to keep BF in peritubular capillaries. Furthermore, research has shown that prolyl hydroxylase controls HIFs, which is how hypoxia-induced transcription works and has helped to reverse the hypoxia effect [60,125].

Glomerular hyperfiltration is a key pathogenetic component of obesity-related glomerulopathy and DM nephropathy [264]. Any additional conditions that enhance metabolic demand, such as anemia and excessive salt intake [265,266], result in relative hypoxia. Patients with MetS experience hypoxia and have an increased CKD and CVD rate [267,268]. Reducing weight, controlling metabolic diseases, reducing glomerular hyperfiltration, and controlling HTN improve and reduce the CKD onset and progression risk.

The principal cause of CKD-associated anemia is interrelated to the relative deficiency of EPO [269]. However, other causes of renal-associated anemia should be considered. As GFR decreases, anemia develops due to blood loss because of frequent unnecessary large-volume blood draws, gastrointestinal bleeding, impaired platelet function, and shortened life span of red blood cells. Measuring plasma levels of EPO can help identify renal anemia. Hence, early identification of the anemia causes and treating them prevent and improve CKD.

Many precipitates of AKI causes lead to low-oxygen availability in the kidneys. Sepsis triggers renal vasoconstriction by releasing endothelin, thereby decreasing regional inner medullary BF [270,271]. Radiocontrast imaging studies increase oxygen consumption because the energy requirements increase for solute reabsorption [19]. Nonsteroidal anti-inflammatory drugs, hemorrhage, decompensated hepatic cirrhosis, CHF, renal artery stenosis, or occlusion induce AKI [243]. Avoiding these agents and early detecting and treating these diseases reduce AKI and CKD risk.

Reduced BF during certain procedures carries a risk of causing AKI. The meta-analysis, clinical follow-up, and epidemiological investigations strongly correlated AKI events and subsequent CKD occurrence [249,250]. It is well documented that AKI severity and frequency predict CKD onset [252]. Therefore, preventive measures to reduce the risk of recurrent AKI will decrease CKD risk.

It was recognized that AKI, because of rhabdomyolysis, cisplatin-containing therapy regimens, ischemia, and radiocontrast agents, reduces oxygen tensions in kidneys (<10 mmHg) in animal studies [96,254]. Similarly, decreased kidney oxygenation was observed in AKI in humans [255]. Notably, renal tissue hypoxia is observed during the early phase of AKI and up to 5 weeks later, following the recovery phase [96,272]. Extended periods of low oxygen levels in the body can reduce pro-angiogenic isoform 164 of VEGF-A manufacture in the kidney. Consequently, dysangiogenic isoforms 120 and 188 are produced in higher quantities [258], leading to a loss of maintenance of the kidney's vascular architecture and a decrease in individual capillaries' size, number, and area [100,258]. Hence, hypoxia prevention and abrupt hypoxia correction help to prevent AKI, which in turn can reduce the onset and progression rate of CKD.

## Conclusions

The kidney is an organ with a high oxygen demand due to its high metabolic activities, fluid, and electrolyte handling. Hypoxia can cause serious kidney damage through various mechanisms, including anemia (anemic hypoxia). Anemia often accompanies CKD, and severe cases can accelerate its onset and progression. Following recommendations to correct Hb levels can prevent the onset of CKD progression and improve life quality; however, the correction of anemia should be conducted cautiously.

Understanding the mechanisms of hypoxia and the associated factors that can precipitate hypoxia in the kidneys such as smoking, diabetes, HTN, obesity, high lipid profile, and OSA are crucial, as attempting to reverse these mechanisms that cause hypoxia and restore oxygen supply can prevent the onset and progression of CKD. To learn more about the relationship between these factors and kidney hypoxia, additional research is needed.

## References

[1] Kovesdy CP (2022). Epidemiology of chronic kidney disease: an update 2022. Kidney Int Suppl.

[2] Inker LA, Grams ME, Levey AS (2019). CKD prognosis consortium. Relationship of estimated GFR and albuminuria to concurrent laboratory abnormalities: An individual participant data meta-analysis in a global consortium. Am J Kidney Dis.

[3] Cappellini MD, Motta I (2015). Anemia in clinical practice—Definition and classification: Does hemoglobin change with aging?. Semin Hematol.

[4] McClellan WM, Flanders WD, Langston RD, Claudine J, Rodney P (2002). Anemia and renal insufficiency are independent risk factors for death among patients with congestive heart failure admitted to community hospitals: a population-based study. J Am Soc Nephrol.

[5] Portolés J, Martín L, Broseta JJ, Cases A (2021). Anemia in chronic kidney disease: From pathophysiology and current treatments, to future agents. Front Med.

[6] Iseki K, Kohagura K (2007). Anemia as a risk factor for chronic kidney disease. Kidney Int Suppl.

[7] Shaikh H, Hashmi MF, Aeddula NR (2023). Anemia of Chronic Renal Disease. https://www.ncbi.nlm.nih.gov/books/NBK539871/..

[8] Chang JM, Chen SC, Huang JC, Su HM, Chen HC (2014). Anemia and left ventricular hypertrophy with renal function decline and cardiovascular events in chronic kidney disease. Am J Med Sci.

[9] Mohanram A, Zhang Z, Shahinfar S, Keane WF, Brenner BM, Toto RD (2004). Anemia and end-stage renal disease in patients with type 2 diabetes and nephropathy. Kidney Int.

[10] Hoshino J, Muenz D, Zee J (2020). Associations of hemoglobin levels with health-related quality of life, physical activity, and clinical outcomes in persons with stage 3-5 nondialysis CKD. J Ren Nutr.

[11] Yi SW, Moon SJ, Yi JJ (2019). Low-normal hemoglobin levels and anemia are associated with increased risk of end-stage renal disease in general populations: A prospective cohort study. PLoS One.

[12] Geng XC, Hu ZP, Lian GY (2015). Erythropoietin ameliorates renal interstitial fibrosis via the inhibition of fibrocyte accumulation. Mol Med Rep.

[13] Binley K, Askham Z, Iqball S (2002). Long-term reversal of chronic anemia using a hypoxia-regulated erythropoietin gene therapy. Blood.

[14] Deicher R, Hörl WH (2003). Anaemia as a risk factor for the progression of chronic kidney disease. Curr Opin Nephrol Hypertens.

[15] Maxwell PH, Osmond MK, Pugh CW (1993). Identification of the renal erythropoietin-producing cells using transgenic mice. Kidney Int.

[16] Johnson DW, Forman C, Vesey DA (2006). Novel renoprotective actions of erythropoietin: new uses for an old hormone. Nephrology.

[17] Patel NS, Kerr-Peterson HL, Brines M (2012). Delayed administration of pyroglutamate helix B surface peptide (pHBSP), a novel nonerythropoietic analog of erythropoietin, attenuates acute kidney injury. Mol Med.

[18] Chattong S, Tanamai J, Kiatsomchai P (2013). Glutaraldehyde erythropoietin protects kidney in ischaemia/reperfusion injury without increasing red blood cell production. Br J Pharmacol.

[19] Fu Q, Colgan SP, Shelley CS (2016). Hypoxia: The force that drives chronic kidney disease. Clin Med Res.

[20] Hu L, Yang C, Zhao T (2012). Erythropoietin ameliorates renal ischemia and reperfusion injury via inhibiting tubulointerstitial inflammation. J Surg Res.

[21] Ardalan MR, Estakhri R, Hajipour B (2013). Erythropoietin ameliorates oxidative stress and tissue injury following renal ischemia/reperfusion in rat kidney and lung. Med Princ Pract.

[22] Mix TC, Brenner RM, Cooper ME (2005). Rationale--Trial to Reduce Cardiovascular Events with Aranesp Therapy (TREAT): evolving the management of cardiovascular risk in patients with chronic kidney disease. Am Heart J.

[23] Inrig JK, Barnhart HX, Reddan D (2012). Effect of hemoglobin target on progression of kidney disease: a secondary analysis of the CHOIR (Correction of Hemoglobin and Outcomes in Renal Insufficiency) trial. Am J Kidney Dis.

[24] Palmer SC, Navaneethan SD, Craig JC (2010). Meta-analysis: erythropoiesis-stimulating agents in patients with chronic kidney disease. Ann Intern Med.

[25] Cody JD, Hodson EM (2016). Recombinant human erythropoietin versus placebo or no treatment for the anaemia of chronic kidney disease in people not requiring dialysis. Cochrane Database Syst Rev.

[26] Covic A, Nistor I, Donciu MD, Dumea R, Bolignano D, Goldsmith D (2014). Erythropoiesis-stimulating agents (ESA) for preventing the progression of chronic kidney disease: a meta-analysis of 19 studies. Am J Nephrol.

[27] Elliott S, Tomita D, Endre Z (2017). Erythropoiesis stimulating agents and reno-protection: a meta-analysis. BMC Nephrol.

[28] Iseki K, Iseki C, Ikemiya Y, Fukiyama K (1996). Risk of developing end-stage renal disease in a cohort of mass screening. Kidney Int.

[29] Tozawa M, Iseki K, Iseki C, Kinjo K, Ikemiya Y, Takishita S (2003). Blood pressure predicts risk of developing end-stage renal disease in men and women. Hypertension.

[30] Iseki K, Ikemiya Y, Iseki C, Takishita S (2003). Haematocrit and the risk of developing end-stage renal disease. Nephrol Dial Transplant.

[31] Farrington DK, Sang Y, Grams ME, Ballew SH, Dunning S, Stempniewicz N, Coresh J (2023). Anemia prevalence, type, and associated risks in a cohort of 5.0 million insured patients in the United States by level of kidney function. Am J Kidney Dis.

[32] Gouva C, Nikolopoulos P, Ioannidis JP, Siamopoulos KC (2004). Treating anemia early in renal failure patients slows the decline of renal function: a randomized controlled trial. Kidney Int.

[33] Kuriyama S, Tomonari H, Yoshida H, Hashimoto T, Kawaguchi Y, Sakai O (1997). Reversal of anemia by erythropoietin therapy retards the progression of chronic renal failure, especially in nondiabetic patients. Nephron.

[34] Singh AK, Szczech L, Tang KL, Barnhart H, Sapp S, Wolfson M, Reddan D (2006). Correction of anemia with epoetin alfa in chronic kidney disease. N Engl J Med.

[35] Drüeke TB, Locatelli F, Clyne N (2006). Normalization of hemoglobin level in patients with chronic kidney disease and anemia. N Engl J Med.

[36] Villar E, Lièvre M, Kessler M (2011). Anemia normalization in patients with type 2 diabetes and chronic kidney disease: results of the NEPHRODIAB2 randomized trial. J Diabetes Complications.

[37] Akaishi M, Hiroe M, Hada Y, Suzuki M, Tsubakihara Y, Akizawa T, KRN321 Study Group (2013). Effect of anemia correction on left ventricular hypertrophy in patients with modestly high hemoglobin level and chronic kidney disease. J Cardiol.

[38] Skali H, Lin J, Pfeffer MA (2013). Hemoglobin stability in patients with anemia, CKD, and type 2 diabetes: an analysis of the TREAT (Trial to Reduce Cardiovascular Events With Aranesp Therapy) placebo arm. Am J Kidney Dis.

[39] Peng B, Kong G, Yang C, Ming Y (2020). Erythropoietin and its derivatives: from tissue protection to immune regulation. Cell Death Dis.

[40] Bahlmann FH, Song R, Boehm SM (2004). Low-dose therapy with the long-acting erythropoietin analogue darbepoetin alpha persistently activates endothelial Akt and attenuates progressive organ failure. Circulation.

[41] Altun B, Yilmaz R, Aki T (2012). Use of mesenchymal stem cells and darbepoetin improve ischemia-induced acute kidney injury outcomes. Am J Nephrol.

[42] Ritz E, Laville M, Bilous RW, O’Donoghue D, Scherhag A, Burger U, de Alvaro F (2007). Correction in Diabetes Study Investigators. Target level for hemoglobin correction in patients with diabetes and CKD: primary results of the Anemia Correction in Diabetes (ACORD) Study. Am J Kidney Dis.

[43] Agarwal R (2005). Hypertension and survival in chronic hemodialysis patients—past lessons and future opportunities. Kidney Int.

[44] Mehdi U, Toto RD (2009). Anemia, diabetes, and chronic kidney disease. Diabetes Care.

[45] Brines M, Cerami A (2012). The receptor that tames the innate immune response. Mol Med.

[46] Brines M, Cerami A (2008). Erythropoietin-mediated tissue protection: reducing collateral damage from the primary injury response. J Intern Med.

[47] Bhutta BS, Alghoula F, Berim I (2023). Hypoxia. StatPearls [Internet].

[48] Watts ER, Walmsley SR (2019). Inflammation and hypoxia: HIF and PHD isoform selectivity. Trends Mol Med.

[49] Hiraga T (2018). Hypoxic microenvironment and metastatic bone disease. Int J Mol Sci.

[50] Evans RG, Smith DW, Lee CJ, Ngo JP, Gardiner BS (2020). What makes the kidney susceptible to hypoxia?. Anat Rec.

[51] Liu ZZ, Bullen A, Li Y, Singh P (2017). Renal oxygenation in the pathophysiology of chronic kidney disease. Front Physiol.

[52] O’Connor PM (2006). Renal oxygen delivery: matching delivery to metabolic demand. Clin Exp Pharmacol Physiol.

[53] Edwards A, Kurtcuoglu V (2022). Renal blood flow and oxygenation. Pflugers Arch.

[54] Evans RG, Ince C, Joles JA, Smith DW, May CN, O'Connor PM, Gardiner BS (2013). Haemodynamic influences on kidney oxygenation: clinical implications of integrative physiology. Clin Exp Pharmacol Physiol.

[55] Ballermann BJ, Obeidat M (2014). Tipping the balance from angiogenesis to fibrosis in CKD. Kidney Int Suppl.

[56] Zhang L, Liu L, Bai M (2020). Hypoxia-induced HE4 in tubular epithelial cells promotes extracellular matrix accumulation and renal fibrosis via NF-κB. FASEB J.

[57] Ponticelli C, Campise MR (2021). The inflammatory state is a risk factor for cardiovascular disease and graft fibrosis in kidney transplantation. Kidney Int.

[58] McCullough PA (2021). Anemia of cardiorenal syndrome. Kidney Int Suppl.

[59] Caravaca-Fontán F, Valladares J, Díaz-Campillejo R, Barroso S, Luna E, Caravaca F (2020). Detrimental effect of renin-angiotensin blockade on progression of chronic kidney disease at later stages: A matter of dosage adjustment? (Article in Spanish). Nefrologia (Engl Ed).

[60] Nangaku M (2006). Chronic hypoxia and tubulointerstitial injury: a final common pathway to end-stage renal failure. J Am Soc Nephrol.

[61] Hui L, Benca R (2021). The bidirectional relationship between obstructive sleep apnea and chronic kidney disease. J Stroke Cerebrovasc Dis.

[62] Brezis M, Rosen S, Silva P (1984). Renal ischemia: a new perspective. Kidney Int.

[63] O’Connor PM, Anderson WP, Kett MM, Evans RG (2006). Renal preglomerular arterial-venous O2 shunting is a structural anti-oxidant defence mechanism of the renal cortex. Clin Exp Pharmacol Physiol.

[64] Chen J, Edwards A, Layton AT (2009). A mathematical model of O2 transport in the rat outer medulla. II. Impact of outer medullary architecture. Am J Physiol Renal Physiol.

[65] Zhang W, Edwards A (2002). Oxygen transport across vasa recta in the renal medulla. Am J Physiol Heart Circ Physiol.

[66] Zhuo JL, Li XC (2013). Proximal nephron. Compr Physiol.

[67] Evans RG, Harrop GK, Ngo JP, Ow CPC, O’Connor PM (2014). Basal renal O2 consumption and the efficiency of O2 utilization for Na+ reabsorption. Am J Physiol Renal Physiol.

[68] Thomson SC, Blantz RC (2008). Glomerulotubular balance, tubuloglomerular feedback, and salt homeostasis. J Am Soc Nephrol.

[69] Palmer LG, Schnermann J (2015). Integrated control of Na transport along the nephron. Clin J Am Soc Nephrol.

[70] Carlström M, Wilcox CS, Arendshorst WJ (2015). Renal autoregulation in health and disease. Physiol Rev.

[71] Balaban RS, Mandel LJ (1988). Metabolic substrate utilization by rabbit proximal tubule. An NADH fluorescence study. Am J Physiol.

[72] Wirthensohn G, Guder WG (1986). Renal substrate metabolism. Physiol Rev.

[73] Epstein FH (1997). Oxygen and renal metabolism. Kidney Int.

[74] Bábíčková J, Klinkhammer BM, Buhl EM (2017). Regardless of etiology, progressive renal disease causes ultrastructural and functional alterations of peritubular capillaries. Kidney Int.

[75] Afsar B, Afsar RE, Dagel T (2018). Capillary rarefaction from the kidney point of view. Clin Kidney J.

[76] Crone C (1963). The permeability of capillaries in various organs as determined by use of the ‘Indicator Diffusion’. Acta Physiol Scand.

[77] Yamamoto T, Tada T, Brodsky SV, Tanaka H, Noiri E, Kajiya F, Goligorsky MS (2002). Intravital videomicroscopy of peritubular capillaries in renal ischemia. Am J Physiol Renal Physiol.

[78] Ivanov KP, Kalinina MK, Levkovich YI (1981). Blood flow velocity in capillaries of brain and muscles and its physiological significance. Microvasc Res.

[79] Mortensen SP, Saltin B (2014). Regulation of the skeletal muscle blood flow in humans. Exp Physiol.

[80] Nippert AR, Biesecker KR, Newman EA (2018). Mechanisms mediating functional hyperemia in the brain. Neuroscientist.

[81] Evans RG, Goddard D, Eppel GA, O'Connor PM (2011). Factors that render the kidney susceptible to tissue hypoxia in hypoxemia. Am J Physiol Regul Integr Comp Physiol.

[82] Lee CJ, Gardiner BS, Ngo JP, Kar S, Evans RG, Smith DW (2017). Accounting for oxygen in the renal cortex: a computational study of factors that predispose the cortex to hypoxia. Am J Physiol Renal Physiol.

[83] Hsu CY, Ordoñez JD, Chertow GM, Fan D, McCulloch CE, Go AS (2008). The risk of acute renal failure in patients with chronic kidney disease. Kidney Int.

[84] Basile DP, Zeng P, Friedrich JL, Yoder MC (2012). Low proliferative potential and impaired angiogenesis of cultured rat kidney endothelial cells. Microcirculation.

[85] Basile DP, Friedrich JL, Spahic J (2011). Impaired endothelial proliferation and mesenchymal transition contribute to vascular rarefaction following acute kidney injury. Am J Physiol Renal Physiol.

[86] Lee CJ, Smith DW, Gardiner BS, Evans RG (2019). Stimulation of erythropoietin release by hypoxia and hypoxemia: similar but different. Kidney Int.

[87] Montero D, Lundby C (2019). Arterial oxygen content regulates plasma erythropoietin independent of arterial oxygen tension: a blinded crossover study. Kidney Int.

[88] Goldfarb-Rumyantzev AS, Alper SL (2014). Short-term responses of the kidney to high altitude in mountain climbers. Nephrol Dial Transplant.

[89] Donnelly S (2003). Why is Erythropoietin Made in the Kidney?. Hypoxia. Advances in Experimental Medicine and Biology.

[90] Eppel GA, Denton KM, Malpas SC, Evans RG (2003). Nitric oxide in responses of regional kidney perfusion to renal nerve stimulation and renal ischaemia. Pflugers Arch.

[91] Edmunds NJ, Marshall JM (2001). Oxygen delivery and oxygen consumption in rat hindlimb during systemic hypoxia: role of adenosine. J Physiol.

[92] Bie P (2009). Blood volume, blood pressure and total body sodium: internal signalling and output control. Acta Physiol.

[93] Evans RG, Bie P (2016). Role of the kidney in the pathogenesis of hypertension: time for a neo-Guytonian paradigm or a paradigm shift?. Am J Physiol Regul Integr Comp Physiol.

[94] Polichnowski AJ, Lan R, Geng H, Griffin KA, Venkatachalam MA, Bidani AK (2014). Severe renal mass reduction impairs recovery and promotes fibrosis after AKI. J Am Soc Nephrol.

[95] Asada N, Takase M, Nakamura J (2011). Dysfunction of fibroblasts of extrarenal origin underlies renal fibrosis and renal anemia in mice. J Clin Invest.

[96] Conde E, Alegre L, Blanco-Sánchez I (2012). Hypoxia inducible factor 1-alpha (HIF-1 alpha) is induced during reperfusion after renal ischemia and is critical for proximal tubule cell survival. PLoS One.

[97] Zager RA, Johnson AC, Becker K (2011). Acute unilateral ischemic renal injury induces progressive renal inflammation, lipid accumulation, histone modification, and “end-stage” kidney disease. Am J Physiol Renal Physiol.

[98] Zager RA, Johnson AC (2009). Renal ischemia-reperfusion injury upregulates histone-modifying enzyme systems and alters histone expression at proinflammatory/profibrotic genes. Am J Physiol Renal Physiol.

[99] Friederich-Persson M, Thörn E, Hansell P, Nangaku M, Levin M, Palm F (2013). Kidney hypoxia, attributable to increased oxygen consumption, induces nephropathy independently of hyperglycemia and oxidative stress. Hypertension.

[100] Kramann R, Tanaka M, Humphreys BD (2014). Fluorescence microangiography for quantitative assessment of peritubular capillary changes after AKI in mice. J Am Soc Nephrol.

[101] Norman JT, Clark IM, Garcia PL (2000). Hypoxia promotes fibrogenesis in human renal fibroblasts. Kidney Int.

[102] Mimura I, Nangaku M (2010). The suffocating kidney: tubulointerstitial hypoxia in end-stage renal disease. Nat Rev Nephrol.

[103] Moeller S, Gioberge S, Brown G (2002). ESRD patients in 2001: global overview of patients, treatment modalities and development trends. Nephrol Dial Transplant.

[104] Gardiner BS, Smith DW, O’Connor PM, Evans RG (2011). A mathematical model of diffusional shunting of oxygen from arteries to veins in the kidney. Am J Physiol Renal Physiol.

[105] Nordsletten DA, Blackett S, Bentley MD, Ritman EL, Smith NP (2006). Structural morphology of renal vasculature. Am J Physiol Heart Circ Physiol.

[106] Welch WJ, Baumgärtl H, Lübbers D, Wilcox CS (2001). Nephron pO2 and renal oxygen usage in the hypertensive rat kidney. Kidney Int.

[107] Olgac U, Kurtcuoglu V (2015). Renal oxygenation: preglomerular vasculature is an unlikely contributor to renal oxygen shunting. Am J Physiol Renal Physiol.

[108] Gardiner BS, Thompson SL, Ngo JP (2012). Diffusive oxygen shunting between vessels in the preglomerular renal vasculature: anatomic observations and computational modeling. Am J Physiol Renal Physiol.

[109] Ngo JP, Kar S, Kett MM (2014). Vascular geometry and oxygen diffusion in the vicinity of artery-vein pairs in the kidney. Am J Physiol Renal Physiol.

[110] Olgac U, Kurtcuoglu V (2016). The Bohr effect is not a likely promoter of renal preglomerular oxygen shunting. Front Physiol.

[111] Lee CJ, Ngo JP, Kar S, Gardiner BS, Evans RG, Smith DW (2017). A pseudo-three-dimensional model for quantification of oxygen diffusion from preglomerular arteries to renal tissue and renal venous blood. Am J Physiol Renal Physiol.

[112] Kuo W, Kurtcuoglu V (2017). Renal arteriovenous oxygen shunting. Curr Opin Nephrol Hypertens.

[113] Ngo JP, Ow CP, Gardiner BS, Kar S, Pearson JT, Smith DW, Evans RG (2016). Diffusive shunting of gases and other molecules in the renal vasculature: physiological and evolutionary significance. Am J Physiol Regul Integr Comp Physiol.

[114] Lee CJ, Gardiner BS, Evans RG, Smith DW (2018). A model of oxygen transport in the rat renal medulla. Am J Physiol Renal Physiol.

[115] Pallone TL, Robertson CR, Jamison RL (1990). Renal medullary microcirculation. Physiol Rev.

[116] O’Connor PM, Evans RG (2010). Structural antioxidant defense mechanisms in the mammalian and nonmammalian kidney: different solutions to the same problem?. Am J Physiol Regul Integr Comp Physiol.

[117] Brezis M, Rosen S (1995). Hypoxia of the renal medulla—its implications for disease. N Engl J Med.

[118] Ren H, Gu L, Andreasen A, Thomsen JS, Cao L, Christensen EI, Zhai XY (2014). Spatial organization of the vascular bundle and the interbundle region: three-dimensional reconstruction at the inner stripe of the outer medulla in the mouse kidney. Am J Physiol Renal Physiol.

[119] Pannabecker TL, Layton AT (2014). Targeted delivery of solutes and oxygen in the renal medulla: role of microvessel architecture. Am J Physiol Renal Physiol.

[120] Fry BC, Edwards A, Sgouralis I, Layton AT (2014). Impact of renal medullary three-dimensional architecture on oxygen transport. Am J Physiol Renal Physiol.

[121] Heyman SN, Rosenberger C, Rosen S (2010). Experimental ischemia-reperfusion: biases and myths—the proximal vs. distal hypoxic tubular injury debate revisited. Kidney Int.

[122] Sgouralis I, Evans RG, Layton AT (2017). Renal medullary and urinary oxygen tension during cardiopulmonary bypass in the rat. Math Med Biol.

[123] Corredor C, Thomson R, Al-Subaie N (2016). Long-term consequences of acute kidney injury after cardiac surgery: a systematic review and meta-analysis. J Cardiothorac Vasc Anesth.

[124] Hu J, Chen R, Liu S (2016). Global incidence and outcomes of adult patients with acute kidney injury after cardiac surgery: a systematic review and meta-analysis. J Cardiothorac Vasc Anesth.

[125] Wang B, Li ZL, Zhang YL, Wen Y, Gao YM, Liu BC (2022). Hypoxia and chronic kidney disease. eBioMedicine.

[126] Koury MJ, Haase VH (2015). Anaemia in kidney disease: harnessing hypoxia responses for therapy. Nat Rev Nephrol.

[127] Nangaku M, Fujita T (2008). Activation of the renin-angiotensin system and chronic hypoxia of the kidney. Hypertens Res.

[128] Liu BC, Tang TT, Lv LL, Lan HY (2018). Renal tubule injury: a driving force toward chronic kidney disease. Kidney Int.

[129] Shaw I, Rider S, Mullins J, Hughes J, Péault B (2018). Pericytes in the renal vasculature: roles in health and disease. Nat Rev Nephrol.

[130] Rabelink TJ, Wijewickrama DC, de Koning EJ (2007). Peritubular endothelium: the Achilles heel of the kidney?. Kidney Int.

[131] Silvestris E, de Pergola G, Rosania R, Loverro G (2018). Obesity as disruptor of the female fertility. Reprod Biol Endocrinol.

[132] Kovesdy CP, Furth SL, Zoccali C (2017). Obesity and kidney disease: hidden consequences of the epidemic. Can J Kidney Health Dis.

[133] Chagnac A, Weinstein T, Korzets A, Ramadan E, Hirsch J, Gafter U (2000). Glomerular hemodynamics in severe obesity. Am J Physiol Renal Physiol.

[134] Tozawa M, Iseki K, Iseki C, Oshiro S, Ikemiya Y, Takishita S (2002). Influence of smoking and obesity on the development of proteinuria. Kidney Int.

[135] Kambham N, Markowitz GS, Valeri AM, Lin J, D'Agati VD (2001). Obesity related glomerulopathy: an emerging epidemic. Kidney Int.

[136] Chagnac A, Weinstein T, Herman M, Hirsh J, Uzi G, Yaacov O (2003). The effects of weight loss on renal function in patients with severe obesity. J Am Soc Nephrol.

[137] Afshinnia F, Wilt TJ, Duval S, Esmaeili A, Ibrahim HN (2010). Weight loss and proteinuria: systematic review of clinical trials and comparative cohorts. Nephrol Dial Transplant.

[138] Hsu CY, McCulloch CE, Iribarren C, Darbinian J, Go AS (2006). Body mass index and risk for end-stage renal disease. Ann Intern Med.

[139] Domrongkitchaiporn S, Sritara P, Kitiyakara C (2005). Risk factors for development of decreased kidney function in a southeast Asian population: a 12-year cohort study. J Am Soc Nephrol.

[140] Shetty S, Parthasarathy S (2015). Obesity hypoventilation syndrome. Curr Pulmonol Rep.

[141] Abuvassin B, Sharma K, Ayas NT, Laher I (2015). Obstructive sleep apnea and kidney disease: a potential bidirectional relationship?. J Clin Sleep Med.

[142] Fleischmann G, Fillafer G, Matterer H, Skrabal F, Kotanko P (2010). Prevalence of chronic kidney disease in patients with suspected sleep apnoea. Nephrol Dial Transplant.

[143] Hanly P (2004). Sleep apnea and daytime sleepiness in end-stage renal disease. Semin Dial.

[144] Prasad R, Jha RK, Keerti A (2022). Chronic kidney disease: its relationship with obesity. Cureus.

[145] Lew QLJ, Allen JC, Nguyen F, Tan NC, Jafar TH (2018). Factors associated with chronic kidney disease and their clinical utility in primary care clinics in a multi-ethnic Southeast Asian population. Nephron.

[146] Palm F (2006). Intrarenal oxygen in diabetes and a possible link to diabetic nephropathy. Clin Exp Pharmacol Physiol.

[147] Stieger N, Worthmann K, Teng B, Engeli S, Das AM, Haller H, Schiffer M (2012). Impact of high glucose and transforming growth factor-β on bioenergetic profiles in podocytes. Metabolism.

[148] Palm F, Buerk DG, Carlsson PO, Hansell P, Liss P (2005). Reduced nitric oxide concentration in the renal cortex of streptozotocin-induced diabetic rats: effects on renal oxygenation and microcirculation. Diabetes.

[149] Sodhi CP, Phadke SA, Batlle D, Sahai A (2001). Hypoxia and high glucose cause exaggerated mesangial cell growth and collagen synthesis: role of osteopontin. Am J Physiol Renal Physiol.

[150] Park CW, Kim JH, Lee JH (2000). High glucose-induced intercellular adhesion molecule-1 (ICAM-1) expression through an osmotic effect in rat mesangial cells is PKC-NF-kappa B-dependent. Diabetologia.

[151] Allen TJ, Cooper ME, Lan HY (2004). Use of genetic mouse models in the study of diabetic nephropathy. Curr Atheroscler Rep.

[152] Cohen MP, Lautenslager GT, Shearman CW (2001). Increased urinary type IV collagen marks the development of glomerular pathology in diabetic d/db mice. Metabolism.

[153] Saleh MA, Pollock DM (2011). Endothelin in Renal Inflammation and Hypertension. Contributions to Nephrology.

[154] Tian XY, Wong WT, Leung FP (2012). Oxidative stress-dependent cyclooxygenase-2-derived prostaglandin f(2α) impairs endothelial function in renovascular hypertensive rats. Antioxid Redox Signal.

[155] Welch WJ (2006). Intrarenal oxygen and hypertension. Clin Exp Pharmacol Physiol.

[156] Tanaka T, Miyata T, Inagi R (2004). Hypoxia in renal disease with proteinuria and/or glomerular hypertension. Am J Pathol.

[157] Welch WJ, Baumgärtl H, Lübbers D, Wilcox CS (2003). Renal oxygenation defects in the spontaneously hypertensive rat: role of AT1 receptors. Kidney Int.

[158] Welch WJ, Blau J, Xie H, Chabrashvili T, Wilcox CS (2005). Angiotensin-induced defects in renal oxygenation: role of oxidative stress. Am J Physiol Heart Circ Physiol.

[159] Eckardt KU, Bernhardt WM, Weidemann A, Warnecke C, Rosenberger C, Wiesener MM, Willam C (2005). Role of hypoxia in the pathogenesis of renal disease. Kidney Int.

[160] Zhu Q, Wang Z, Xia M (2011). Silencing of hypoxia-inducible factor-1α gene attenuated angiotensin II-induced renal injury in Sprague-Dawley rats. Hypertension.

[161] Heidland A, Gerabek W, Sebekova K (2001). Franz Volhard and Theodor Fahr: achievements and controversies in their research in renal disease and hypertension. J Hum Hypertens.

[162] Hansell P, Welch WJ, Blantz RC, Palm F (2013). Determinants of kidney oxygen consumption and their relationship to tissue oxygen tension in diabetes and hypertension. Clin Exp Pharmacol Physiol.

[163] Simão S, Gomes P, Pinto V (2011). Age-related changes in renal expression of oxidant and antioxidant enzymes and oxidative stress markers in male SHR and WKY rats. Exp Gerontol.

[164] Panico C, Luo Z, Damiano S (2009). Renal proximal tubular reabsorption is reduced in adult spontaneously hypertensive rats roles of superoxide and Na+/H+ exchanger 3. Hypertension.

[165] Economides PA, Caselli A, Zuo CS (2004). Kidney oxygenation during water diuresis and endothelial function in patients with type 2 diabetes and subjects at risk to develop diabetes. Metabolism.

[166] Darouich S, Goucha R, Jaafoura MH, Zekri S, Maiz HB, Kheder A (2011). Clinicopathological characteristics of obesity-associated focal segmental glomerulosclerosis. Ultrastruct Pathol.

[167] Michelakis ED, Hampl V, Nsair A (2002). Diversity in mitochondrial function explains differences in vascular oxygen sensing. Circ Res.

[168] Favreau F, Rossard L, Zhang K (2009). Expression and modulation of translocator protein and its partners by hypoxia reoxygenation or ischemia and reperfusion in porcine renal models. Am J Physiol Renal Physiol.

[169] Lecanu L, Yao ZX, McCourty A, Sidahmed EK, Orellana ME, Burnier MN, Papadopoulos V (2013). Control of hypercholesterolemia and atherosclerosis using the cholesterol recognition/interaction amino acid sequence of the translocator protein TSPO. Steroids.

[170] Lassance L, Haghiac M, Minium J, Catalano P, Mouzon SH (2015). Obesity-induced down-regulation of the mitochondrial translocator protein (TSPO) impairs placental steroid production. J Clin Endocrinol Metab.

[171] Massy ZA, Keane WF (1996). Pathogenesis of atherosclerosis. Semin Nephrol.

[172] Gloviczki ML, Saad A, Textor SC (2013). Blood oxygen level-dependent (BOLD) MRI analysis in atherosclerotic renal artery stenosis. Curr Opin Nephrol Hypertens.

[173] Warner L, Gomez SI, Bolterman R, Haas JA, Bentley MD, Lerman LO, Romero JC (2009). Regional decreases in renal oxygenation during graded acute renal arterial stenosis: a case for renal ischemia. Am J Physiol Regul Integr Comp Physiol.

[174] Evans RG, Eppel GA, Anderson WP, Denton KA (2004). Mechanisms underlying the differential control of blood flow in the renal medulla and cortex. J Hypertens.

[175] Iliescu R, Fernandez SR, Kelsen S, Maric C, Chade AR (2010). Role of renal microcirculation in experimental renovascular disease. Nephrol Dial Transplant.

[176] Textor SC, Lerman L (2010). Renovascular hypertension and ischemic nephropathy. Am J Hypertens.

[177] Gloviczki ML, Keddis MT, Garovic VD (2013). TGF expression and macrophage accumulation in atherosclerotic renal artery stenosis. Clin J Am Soc Nephrol.

[178] Novick AC, Pohl MA, Schreiber M, Vidt DG (1983). Revascularization for preservation of renal function in patients with atherosclerotic renovascular disease. J Urol.

[179] Mistry S, Ives N, Harding J (2007). Angioplasty and STent for Renal Artery Lesions (ASTRAL trial): rationale, methods and results so far. J Hum Hypertens.

[180] Murphy TP, Cooper CJ, Dworkin LD (2005). The Cardiovascular Outcomes with Renal Atherosclerotic Lesions (CORAL) study: rationale and methods. J Vasc Interv Radiol.

[181] Whelton PK, Carey RM, Aronow WS (2018). 2017 ACC/AHA/AAPA/ABC/ACPM/AGS/APhA/ASH/ASPC/NMA/PCNA Guideline for the Prevention, Detection, Evaluation, and Management of High Blood Pressure in Adults: Executive Summary: A Report of the American College of Cardiology/American Heart Association Task Force on Clinical Practice Guidelines. Hypertension.

[182] Noborisaka Y, Ishizaki M, Yamada Y, Honda R, Yokoyama H, Miyao M, Tabata M (2013). The effects of continuing and discontinuing smoking on the development of chronic kidney disease (CKD) in the healthy middle-aged working population in Japan. Environ Health Prev Med.

[183] García-Esquinas E, Loeffler LF, Weaver VM, Fadrowski JJ, Navas-Acien A (2013). Kidney function and tobacco smoke exposure in US adolescents. Pediatrics.

[184] Huang MF, Lin WL, Ma YC (2005). A study of reactive oxygen species in mainstream of cigarette. Indoor Air.

[185] Ejerblad E, Fored CM, Lindblad P (2004). Association between smoking and chronic renal failure in a nationwide population-based case-control study. J Am Soc Nephrol.

[186] Yacoub R, Habib H, Lahdo A (2010). Association between smoking and chronic kidney disease: a case control study. BMC Public Health.

[187] Hallan SI, Orth SR (2011). Smoking is a risk factor in the progression to kidney failure. Kidney Int.

[188] Orth SR (2004). Effects of smoking on systemic and intrarenal hemodynamics: influence on renal function. J Am Soc Nephrol.

[189] Jaimes EA, Tian RX, Raij L (2007). Nicotine: the link between cigarette smoking and the progression of renal injury?. Am J Physiol Heart Circ Physiol.

[190] Rezonzew G, Chumley P, Feng W, Hua P, Siegal GP, Jaimes EA (2012). Nicotine exposure and the progression of chronic kidney disease: role of the α7-nicotinic acetylcholine receptor. Am J Physiol Renal Physiol.

[191] Jain G, Jaimes EA (2013). Nicotine signaling and progression of chronic kidney disease in smokers. Biochem Pharmacol.

[192] Arany I, Reed DK, Grifoni SC, Chandrashekar K, Booz GW, Juncos LA (2012). A novel U-STAT3-dependent mechanism mediates the deleterious effects of chronic nicotine exposure on renal injury. Am J Physiol Renal Physiol.

[193] Urbanetti JS (1981). Carbon monoxide poisoning. Prog Clin Biol Res.

[194] Badman DG, Jaffé ER (1996). Blood and air pollution; state of knowledge and research needs. Otolaryngol Head Neck Surg.

[195] Brook RD, Brook JR, Urch B, Vincent R, Rajagopalan S, Silverman F (2002). Inhalation of fine particulate air pollution and ozone causes acute arterial vasoconstriction in healthy adults. Circulation.

[196] Rundell KW, Hoffman JR, Caviston R, Bulbulian R, Hollenbach AM (2007). Inhalation of ultrafine and fine particulate matter disrupts systemic vascular function. Inhal Toxicol.

[197] van Eeden SF, Tan WC, Suwa T (2001). Cytokines involved in the systemic inflammatory response induced by exposure to particulate matter air pollutants (PM(10)). Am J Respir Crit Care Med.

[198] Wang XR, Christiani DC (2000). Respiratory symptoms and functional status in workers exposed to silica, asbestos, and coal mine dusts. J Occup Environ Med.

[199] Wang XR, Zhang HX, Sun BX (2005). A 20-year follow-up study on chronic respiratory effects of exposure to cotton dust. Eur Respir J.

[200] Young T, Palta M, Dempsey J, Skatrud J, Weber S, Badr S (1993). The occurrence of sleep-disordered breathing among middle-aged adults. N Engl J Med.

[201] Kanbay A, Buyukoglan H, Ozdogan N (2012). Obstructive sleep apnea syndrome is related to the progression of chronic kidney disease. Int Urol Nephrol.

[202] Sakaguchi Y, Shoji T, Kawabata H (2011). High prevalence of obstructive sleep apnea and its association with renal function among nondialysis chronic kidney disease patients in Japan: a cross-sectional study. Clin J Am Soc Nephrol.

[203] Nicholl DDM, Ahmed SB, Loewen AHS (2012). Declining kidney function increases the prevalence of sleep apnea and nocturnal hypoxia. Chest.

[204] Hanly PJ, Ahmed SB (2014). Sleep apnea and the kidney: is sleep apnea a risk factor for chronic kidney disease?. Chest.

[205] Foster GE, Poulin MJ, Hanly PJ (2007). Intermittent hypoxia and vascular function: implications for obstructive sleep apnoea. Exp Physiol.

[206] Lavie L (2003). Obstructive sleep apnoea syndrome--an oxidative stress disorder. Sleep Med Rev.

[207] Pialoux V, Hanly PJ, Foster GE (2009). Effects of exposure to intermittent hypoxia on oxidative stress and acute hypoxic ventilatory response in humans. Am J Respir Crit Care Med.

[208] Yuan G, Khan SA, Luo W, Nanduri J, Semenza GL, Prabhakar NR (2011). Hypoxia-inducible factor 1 mediates increased expression of NADPH oxidase-2 in response to intermittent hypoxia. J Cell Physiol.

[209] Sun W, Yin X, Wang Y (2013). Intermittent hypoxia-induced renal antioxidants and oxidative damage in male mice: hormetic dose response. Dose Response.

[210] Gilmartin GS, Lynch M, Tamisier R, Weiss JW (2010). Chronic intermittent hypoxia in humans during 28 nights results in blood pressure elevation and increased muscle sympathetic nerve activity. Am J Physiol Heart Circ Physiol.

[211] Zalucky AA, Nicholl DD, Hanly PJ (2015). Nocturnal hypoxemia severity and renin-angiotensin system activity in obstructive sleep apnea. Am J Respir Crit Care Med.

[212] Aldigier JC, Kanjanbuch T, Ma LJ, Brown NJ, Fogo AB (2005). Regression of existing glomerulosclerosis by inhibition of aldosterone. J Am Soc Nephrol.

[213] Morgan BJ (2007). Vascular Consequences of Intermittent Hypoxia. Hypoxia and the Circulation. Advances in Experimental Medicine and Biology.

[214] Zoccali C, Mallamaci F, Tripepi G, Benedetto FA (2001). Autonomic neuropathy is linked to nocturnal hypoxaemia and to concentric hypertrophy and remodelling in dialysis patients. Nephrol Dial Transplant.

[215] Quercioli A, Mach F, Montecucco F (2010). Inflammation accelerates atherosclerotic processes in obstructive sleep apnea syndrome (OSAS). Sleep Breath.

[216] Garvey JF, Taylor CT, McNicholas WT (2009). Cardiovascular disease in obstructive sleep apnoea syndrome: the role of intermittent hypoxia and inflammation. Eur Respir J.

[217] Zou AP, Yang ZZ, Li PL, Cowley AW (2001). Oxygen-dependent expression of hypoxia-inducible factor-1 alpha in renal medullary cells of rats. Physiol Genomics.

[218] Béguin PC, Belaidi E, Godin-Ribuot D, Lévy P, Ribuot C (2007). Intermittent hypoxia-induced delayed cardioprotection is mediated by PKC and triggered by p38 MAP kinase and Erk1/2. J Mol Cell Cardiol.

[219] Nangaku M, Rosenberger C, Heyman SN, Eckardt KU (2013). Regulation of hypoxia-inducible factor in kidney disease. Clin Exp Pharmacol Physiol.

[220] Miraliakbari R, Francalancia NA, Lust RM, Gerardo JA, Ng PC, Sun YS, Chitwood WR (2000). Differences in myocardial and peripheral VEGF and KDR levels after acute ischemia. Ann Thorac Surg.

[221] Kaneko T, Shimizu A, Mii A (2012). Role of matrix metalloproteinase-2 in recovery after tubular damage in acute kidney injury in mice. Nephron Exp Nephrol.

[222] Rosenberger C, Rosen S, Shina A (2006). Hypoxia-inducible factors and tubular cell survival in isolated perfused kidneys. Kidney Int.

[223] Leonard MO, Kieran NE, Howell K (2006). Reoxygenation-specific activation of the antioxidant transcription factor Nrf2 mediates cytoprotective gene expression in ischemia-reperfusion injury. FASEB J.

[224] Uchida T, Rossignol F, Matthay MA, Mounier R, Couette S, Clottes E, Clerici C (2004). Prolonged hypoxia differentially regulates hypoxia-inducible factor (HIF)-1alpha and HIF-2alpha expression in lung epithelial cells: implication of natural antisense HIF-1alpha. J Biol Chem.

[225] Yang ZZ, Zhang AY, Yi FX, Li PL, Zou Zou (2003). Redox regulation of HIF-1alpha levels and HO-1 expression in renal medullary interstitial cells. Am J Physiol Renal Physiol.

[226] Zou AP, Cowley AW Jr (2003). Reactive oxygen species and molecular regulation of renal oxygenation. Acta Physiol Scand.

[227] Schulz R, Schmidt D, Blum A (2000). Decreased plasma levels of nitric oxide derivatives in obstructive sleep apnoea: response to CPAP therapy. Thorax.

[228] Hayflick JS, Kilgannon P, Gallatin WM (1998). The intercellular adhesion molecule (ICAM) family of proteins: new members and novel functions. Immunol Res.

[229] Ates E, Yalcin AU, Yilmaz S, Koken T, Tokyol C (2005). Protective effect of erythropoietin on renal ischemia and reperfusion injury. ANZ J Surg.

[230] Manotham K, Tanaka T, Matsumoto M (2004). Transdifferentiation of cultured tubular cells induced by hypoxia. Kidney Int.

[231] Dore-Duffy P, Balabanov R, Beaumont T, Hritz MA, Harik SI, LaManna JC (1999). Endothelial activation following prolonged hypobaric hypoxia. Microvasc Res.

[232] Malaponte G, Bevelacqua V, Fatuzzo P, Rapisarda F, Emmanuele G, Travali S, Mazzarino MC (2002). IL-1beta, TNF-alpha and IL-6 release from monocytes in haemodialysis patients in relation to dialytic age. Nephrol Dial Transplant.

[233] Sean Eardley K, Cockwell P (2005). Macrophages and progressive tubulointerstitial disease. Kidney Int.

[234] Zager RA, Johnson AC, Andress D, Becker K (2013). Progressive endothelin-1 gene activation initiates chronic/end-stage renal disease following experimental ischemic/reperfusion injury. Kidney Int.

[235] Luscinskas FW, Ma S, Nusrat A, Parkos CA, Shaw SK (2002). Leukocyte transendothelial migration: a junctional affair. Semin Immunol.

[236] Konstantopoulos K, Hanley WD, Wirtz D (2003). Receptor-ligand binding: ‘catch’ bonds finally caught. Curr Biol.

[237] Manjunath N, Correa M, Ardman M, Ardman B (1995). Negative regulation of T-cell adhesion and activation by CD43. Nature.

[238] Van der Vieren M, Le Trong H, Wood CL, Moore PF, John TS, Staunton DE, Gallatin WM (1995). A novel leukointegrin, alpha d beta 2, binds preferentially to ICAM-3. Immunity.

[239] Clark MC, Baum LG (2012). T cells modulate glycans on CD43 and CD45 during development and activation, signal regulation, and survival. Ann N Y Acad Sci.

[240] Shelley CS, Da Silva N, Teodoridis JM (2001). During U937 monocytic differentiation repression of the CD43 gene promoter is mediated by the single-stranded DNA binding protein Pur alpha. Br J Haematol.

[241] Lameire N, Van Biesen W, Vanholder R (2005). Acute renal failure. Lancet.

[242] Cho AY, Oh JH, Oh SS, Lee KY, Sun IO (2023). Clinical characteristics of acute kidney injury in patients with glyphosate surfactant herbicide poisoning. Kidney Res Clin Pract.

[243] Klenzak J, Himmelfarb J (2005). Sepsis and the kidney. Crit Care Clin.

[244] Regueira T, Andresen M, Mercado M, Downey P (2011). Physiopathology of acute renal failure during sepsis. Med Intensiva.

[245] Helling TS (2002). Ruminations of an ordinary hepatic surgeon: a journey through the pitfalls of major liver resections. J Gastrointest Surg.

[246] Takahama T (2001). Post operative complications after hepatopancreatoduodenectomy (HPD). (Article in Japanese). Nihon Geka Gakkai Zasshi.

[247] Carmichael P, Carmichael AR (2003). Acute renal failure in the surgical setting. ANZ J Surg.

[248] Askenazi DJ, Feig DI, Graham NM, Hui-Stickle S, Goldstein SL (2006). 3-5 year longitudinal follow-up of pediatric patients after acute renal failure. Kidney Int.

[249] Coca SG, Singanamala S, Parikh CR (2012). Chronic kidney disease after acute kidney injury: a systematic review and meta-analysis. Kidney Int.

[250] Wald R, Quinn RR, Luo J (2009). Chronic dialysis and death among survivors of acute kidney injury requiring dialysis. JAMA.

[251] Bucaloiu ID, Kirchner HL, Norfolk ER, Hartle JE, Perkins RM (2012). Increased risk of death and de novo chronic kidney disease following reversible acute kidney injury. Kidney Int.

[252] Chawla LS, Amdur RL, Amodeo S, Kimmel PL, Palant CE (2011). The severity of acute kidney injury predicts progression to chronic kidney disease. Kidney Int.

[253] Belayev LY, Palevsky PM (2014). The link between acute kidney injury and chronic kidney disease. Curr Opin Nephrol Hypertens.

[254] Abdelkader A, Ho J, Ow CP, Eppel GA, Rajapakse NW, Schlaich MP, Evans RG (2014). Renal oxygenation in acute renal ischemia-reperfusion injury. Am J Physiol Renal Physiol.

[255] Redfors B, Bragadottir G, Sellgren J, Kristina S, Sven-Erik R (2010). Acute renal failure is NOT an “acute renal success”-a clinical study on the renal oxygen supply/demand relationship in acute kidney injury. Crit Care Med.

[256] Yingjie K, Haihong Y, Lingwei C (2019). Apoptosis repressor with caspase recruitment domain deficiency accelerates ischemia/reperfusion (I/R)-induced acute kidney injury by suppressing inflammation and apoptosis: The role of AKT/mTOR signaling. Biomed Pharmacother.

[257] Chang FC, Chou YH, Chen YT, Lin SL (2012). Novel insights into pericyte-myofibroblast transition and therapeutic targets in renal fibrosis. J Formos Med Assoc.

[258] Lin SL, Chang FC, Schrimpf C (2011). Targeting endothelium-pericyte cross talk by inhibiting VEGF receptor signaling attenuates kidney microvascular rarefaction and fibrosis. Am J Pathol.

[259] Menshikh A, Scarfe L, Delgado R, Finney C, Zhu Y, Yang H, de Caestecker MP (2019). Capillary rarefaction is more closely associated with CKD progression after cisplatin, rhabdomyolysis, and ischemia-reperfusion-induced AKI than renal fibrosis. Am J Physiol Renal Physiol.

[260] Kong T, Eltzschig HK, Karhausen J, Colgan SP, Shelley CS (2004). Leukocyte adhesion during hypoxia is mediated by HIF-1-dependent induction of beta2 integrin gene expression. Proc Natl Acad Sci USA.

[261] Wang J, Shen F, Liu F, Zhuang S (2022). Histone modifications in acute kidney injury. Kidney Dis (Basel).

[262] Basile DP, Donohoe D, Roethe K, Osborn JL (2001). Renal ischemic injury results in permanent damage to peritubular capillaries and influences long-term function. Am J Physiol Renal Physiol.

[263] Csiki DM, Ababneh H, Tóth A (2023). Hypoxia-inducible factor activation promotes osteogenic transition of valve interstitial cells and accelerates aortic valve calcification in a mice model of chronic kidney disease. Front Cardiovasc Med.

[264] Brenner BM, Meyer TW, Hostetter TH (1982). Dietary protein intake and the progressive nature of kidney disease: the role of hemodynamically mediated glomerular injury in the pathogenesis of progressive glomerular sclerosis in aging, renal ablation, and intrinsic. N Engl J Med.

[265] Krikken JA, Lely AT, Bakker SJ, Navis G (2007). The effect of a shift in sodium intake on renal hemodynamics is determined by body mass index in healthy young men. Kidney Int.

[266] Ritz E (2007). Lowering salt intake-an important strategy in the management of renal disease. Nat Clin Pract Nephrol.

[267] Chen J, Muntner P, Hamm LL (2004). The metabolic syndrome and chronic kidney disease in U.S. adults. Ann Intern Med.

[268] Thomas G, Sehgal AR, Kashyap SR, Srinivas TR, Kirwan JP, Navaneethan S (2011). Metabolic syndrome and kidney disease: A systematic review and meta-analysis. Clin J Am Soc Nephrol.

[269] Lederer E, Ouseph R (2007). Chronic kidney disease. Am J Kidney Dis.

[270] Weisbord SD, Palevsky PM (2005). Radiocontrast-induced acute renal failure. J Intensive Care Med.

[271] Heyman SN, Rosenberger C, Rosen S (2005). Regional alterations in renal haemodynamics and oxygenation: a role in contrast medium-induced nephropathy. Nephrol Dial Transplant.

[272] Rosenberger C, Pratschke J, Rudolph B (2007). Immunohistochemical detection of hypoxia-inducible factor-1alpha in human renal allograft biopsies. J Am Soc Nephro.

